# Efficient PD-L1 imaging of murine glioblastoma with FUS-aided immunoPET by leveraging FcRn-antibody interaction

**DOI:** 10.7150/thno.87168

**Published:** 2023-10-16

**Authors:** Céline Chevaleyre, Anthony Novell, Nicolas Tournier, Ambre Dauba, Steven Dubois, Dimitri Kereselidze, Erwan Selingue, Benoit Jego, Bernard Maillère, Benoit Larrat, Hervé Nozach, Charles Truillet

**Affiliations:** 1 Paris-Saclay University, CEA, CNRS, Inserm, BioMaps, Service Hospitalier Frédéric Joliot, Orsay France.; 2 Paris-Saclay University, CEA, INRAE, Medicines and Healthcare Technologies Department, SIMoS, Gif-sur-Yvette, France.; 3 Paris-Saclay University, CEA, CNRS, NeuroSpin/BAOBAB, Centre d'études de Saclay, Bâtiment 145, 91191 Gif sur Yvette, France.

**Keywords:** ImmunoPET, therapeutic ultrasound, Fc receptor, neonatal, PD-L1, Immune Checkpoint Inhibitors, Glioblastoma

## Abstract

**Rationale:** The passage of antibodies through the blood-brain barrier (BBB) and the blood-tumoral barrier (BTB) is determinant not only to increase the immune checkpoint inhibitors efficacy but also to monitor prognostic and predictive biomarkers such as the programmed death ligand 1 (PD-L1) via immunoPET. Although the involvement of neonatal Fc receptor (FcRn) in antibody distribution has been demonstrated, its function at the BBB remains controversial, while it is unknown at the BTB. In this context, we assessed FcRn's role by pharmacokinetic immunoPET imaging combined with focused ultrasounds (FUS) using unmodified and FcRn low-affinity IgGs targeting PD-L1 in a preclinical orthotopic glioblastoma model.

**Methods:** Transcranial FUS were applied over the whole brain in mice shortly before injecting the anti-PD-L1 IgG ^89^Zr-DFO-C4 or its FcRn low-affinity mutant ^89^Zr-DFO-C4^Fc-*MUT*^ in a syngeneic glioblastoma murine model (GL261-GFP). Brain uptake was measured from PET scans acquired up to 7 days post-injection. Kinetic modeling was performed to compare the brain kinetics of both C4 formats.

**Results:** FUS efficiently enhanced the delivery of both C4 radioligands in the brain with high reproducibility. ^89^Zr-DFO-C4^Fc-*MUT*^ mean concentrations in the brain reached a significant uptake of 3.75±0.41%ID/cc with FUS against 1.92±0.45%ID/cc without, at 1h post-injection. A substantial and similar entry of both C4 radioligands was observed at a rate of 0.163±0.071 mL/h/g of tissue during 10.4±4.6min. The impaired interaction with FcRn of ^89^Zr-DFO-C4^Fc-*MUT*^ significantly decreased the efflux constant from the healthy brain tissue to plasma compared with non-mutated IgG. Abolishing FcRn interaction allows determining the target engagement related to the specific binding as soon as 12h post-injection.

**Conclusion:** Abolishing Fc-FcRn interaction confers improved kinetic properties to ^89^Zr-DFO-C4^Fc-MUT^ for immunoPET imaging. FUS-aided BBB/BTB disruption enables quantitative imaging of PD-L1 expression by glioblastoma tumors within the brain.

## Introduction

Patients diagnosed with glioblastoma (GBM) have a harrowing overall survival of 13 to 16 months following the standard-of-care treatments 1. GBM is the most common primary brain tumor and is highly aggressive. Immunotherapies have the possibility to be a keystone in eradicating GBM as their microenvironment is immunosuppressive. Ex vivo staining of biopsies have shown that the immune checkpoint programmed death ligand 1 (PD-L1) is expressed by most GBM neoplastic tissues, which suggests a promising role for immune checkpoints inhibitors (ICI) such as anti-PD(L)1 antibodies 2. However, the efficacy of ICI in treating brain malignancies such as GBM has been proven insufficient to improve patient overall survival 3-5. Durations of response were nevertheless greater in patients treated with the anti-PD1 antibody nivolumab compared to those treated with the standard-of-care in the Checkmate 143 trial (NCT02017717) 4. Clinical trials notably informed on the need for an effective minimally-invasive method to select patients who will benefit from ICI and monitor the immune response at the central nervous system (CNS) level.

PD-L1 expression by neoplastic tissues or its microenvironment is the most predictive biomarker of anti-PD(L)1 therapy response in most tumors 6,7. It has not been investigated in GBM as PD-L1 expression is usually evaluated by immunochemistry on tumor biopsies. Moreover, PD-L1 expression is known to be spatially heterogeneous and to change over time with treatments 8. Repeated biopsies over the therapy course are neither always feasible nor acceptable, particularly for CNS localizations. In this regard, non-invasive detection of biomarkers by immunoPET has emerged as a powerful tool to monitor response to ICI therapy. ImmunoPET imaging using radiolabeled antibodies targeting PD-(L)1 allows quantification and the *in vivo* assessment of the inter- and intratumoral heterogeneity of the biomarker expression 9. However, immunoPET imaging within the CNS remains challenging due to the inability of antibodies to cross the blood-brain barrier (BBB). Niemeijer *et al.* and Nienhuis *and al.* obtained uneven accumulation between lesions of the anti-PD1 antibody ^89^Zr-nivolumab or ^18^F-adnectins targeting PD-L1 in brain metastases of patients 9,10. This uptake in some brain metastases but not all within a patient probably reflects the tumor-induced loss in the integrity of the BBB rather than the local expression of targeted biomarkers. It is therefore essential to propose strategies to overcome the BBB and improve the brain kinetics of radiolabeled antibodies to enable quantitative estimation of immune biomarkers such as PD-L1 in infiltrative brain tumors like GBM using immunoPET 11.

The engineering of antibodies is currently investigated to improve their brain delivery 12. This includes modulation of neonatal Fc receptor (FcRn) mediated transcytosis. Reducing the affinity of an antibody for the FcRn has notably been proposed to optimize the peripheral and brain kinetics of radiolabeled antibodies for immunoPET 13. According to the tissue considered, FcRn is responsible for the recycling and transcytosis of the Fc-containing proteins 14. FcRn's physiological function is to bind endogenous immunoglobulin G (IgG) and albumin at acidic pH to protect them from lysosomal degradation, maintaining their serum homeostasis 15. The loss of affinity for the FcRn obtained by substituting two key amino acids of the Fc-domain of an IgG (H310A and H435Q) decreases the plasma half-life of antibodies 16,17. Besides the advantages for PET imaging, the affinity loss for the FcRn could impact IgG brain distribution. FcRn is highly expressed by the CNS endothelium 18. Although there has yet to be a consensus regarding the role of FcRn in controlling IgG transport across brain endothelial cells. Some studies on the brain distribution of IgG in FcRn knock-out mice led to the conclusion of an absence of a FcRn mediated transcytosis at the BBB 19,20. However, those studies are subject to discussion due to potential compensatory mechanisms associated with FcRn depletion. Moreover, some evidence supports a FcRn-mediated efflux of IgG from the brain to the circulation. After intracranial injection, brain clearance of IgG with improved FcRn affinity was faster compared with IgG with reduced FcRn affinity 21. This property was used effectively to promote IgG-mediated amyloid plaque removal in a mouse model of Alzheimer's disease 22. To our knowledge, the particular role of FcRn at the blood-tumoral barrier (BTB) has never been investigated.

Transcranial application of low-intensity focused ultrasound (FUS) with the injection of microbubbles (MB) was shown to enable the brain delivery of drugs by reversibly disrupting the BBB. Local mechanic and shear stress induced by the oscillations of MB (alternation of expansion and compression of the gaseous core of the MB) loosen the tight junctions and increase the pinocytic activity of endothelial cells 23,24. FUS are investigated in preclinical models and clinical trials to treat numerous CNS diseases, notably GBM 25-27. FUS-aided immunoPET was useful in demonstrating the increased brain delivery of radiolabeled antibodies from a therapeutic perspective 28,29. Meng et al. reported enhanced delivery of trastuzumab across the BBB with magnetic resonance-guided FUS in patients with HER2-positive breast cancer and brain metastases. In this study, the HER2-positive status of the brain metastases was determined on surgical specimens previously collected at different temporality before treatments 30. The NCT05879120 clinical trial aims to estimate the median overall survival of patients with recurrent GBM with FUS BBB opening and neo-adjuvant pembrolizumab 31. The inclusion criteria of patients in this study do not include the determination of the brain tumors' PD(L)1 status. FUS-aided immunoPET could provide a companion theranostic approach to assess the likelihood of response of brain tumors to immunotherapy.

**Scheme 1:**
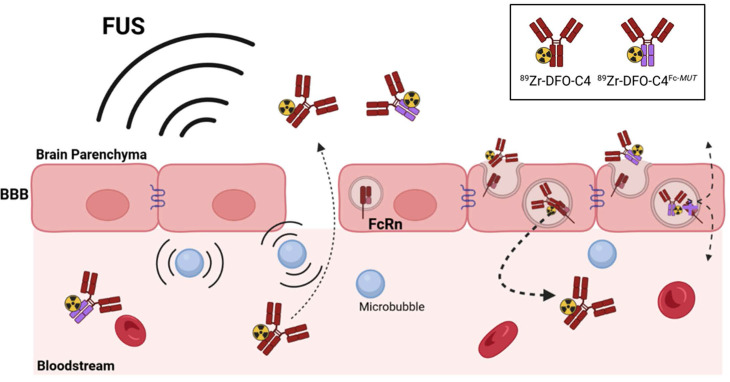
Scheme describing the rational of focused ultrasound to deliver through the blood-brain barrier a low FcRn affinity IgG in the brain parenchyma for immunoPET imaging. *The disruption of the tight junctions of endothelial cells allows the paracellular diffusion of antibodies to the brain parenchyma. The loss of affinity for the FcRn should modify the fate of the radiolabeled C4 antibody targeting PD-L1.*

Here, we aimed to investigate the potential of FUS-aided immunoPET to provide quantitative imaging of PD-L1 expression by glioblastoma tumors within the brain. The human recombinant IgG1, C4, targeting human and murine PD-L1 and its engineered low FcRn affinity (H310A/H435Q) mutant (C4^Fc-*MUT*^) were used to perform FUS-aided immunoPET in a syngeneic glioblastoma murine model (**Scheme 1**). Kinetic modeling was performed to interpret brain PET data, estimate PD-L1 expression, and elucidate the importance of FcRn function on the transport of the radiolabeled antibody across the BBB/BTB.

## Methods

**Production of the C4 ligands.** C4 is a human recombinant IgG1 cross-reacting with human and murine PD-L1 32. The C4 ligands production has been performed as already described in Bouleau *et al.* 17. Briefly, they were obtained by transient transfection of HEK293 FreestyleTM cells (Thermo-Fisher) with AbVec2.0-IGHG1 and AbVec1.1-IGLC plasmids corresponding to the IgG C4 heavy and light chains, respectively. After transient cell transfection, the supernatant was collected and purified using Lambda FabSelect columns (GE Healthcare).

**FcRn binding assay.** IgG/human FcRn and IgG/murine FcRn affinity were measured using the Lumit™ FcRn Binding Immunoassay kit (Promega) following the protocol described by Nath *et al.*^34^. To measure IgG/mFcRn interaction, hFcRn was substituted by recombinant mFcRn with terminus biotin (ACROBiosystems). The recombinant mFcRn was introduced at a concentration of 0.25 mg/mL. Experiments were run in triplicate. Normalized luminescence data were generated by assigning 100% to the maximum bioluminescent signal obtained in the absence of IgG.

**Radiolabeling.** The anti-PD-L1 C4 ligands were radiolabeled according to a previously published protocol 17. First, the p-isothiocyanatobenzyldesferrioxamine (p-NCS-Bz-DFO, Macrocyclics) was conjugated to the C4, and then the radiolabeling with ^89^Zr-oxalate (PerkinElmer) was performed. After the purification of the DFO-anti-PD-L1 ligands with a PD-10 column (GE Healthcare), DFO-anti-PD-L1 ligands were incubated with [^89^Zr]Zr-oxalate for 1 h at 37 °C. The ^89^Zr-labeled DFO-anti-PD-L1 ligand conjugates were then purified with a PD-10 column and buffer exchanged in HEPES solution (Gibco) with a Vivaspin centrifugal concentrator (Sartorius). Radiochemical purity was assessed by instant thin-layer chromatography (iTLC) and high-performance liquid chromatography (HPLC) analyses (Figure S1).

**Cell culture.** Murine glioma's cell line GL261 transfected to produce Green Fluorescent Protein (GL261-GFP) were obtained from the Institute of Neurophysiopathology, Aix-Marseille University. Cells were cultured in Dulbecco's Modified Eagle Medium (Gibco) supplemented with 10 % heat-inactivated fetal bovine serum and 1% penicillin-streptomycin (Gibco) at 37 °C in a humidified atmosphere containing 95% air and 5% carbon dioxide.

**Animals.** Animal experiments were performed on six weeks old female C57BL/6 NRj mice (Janvier Labs). Animal experiments were performed according to the European Directive 2010/63/EU and its transposition into French law (Decree No. 2013-118). The research project was conducted at the CEA-SHFJ imaging platform (authorization D91-471-105) and was approved by a local ethics committee (CETEA-CEA DSV IdF). Mice were housed in standard conditions (microisolator polycarbonate cages, aspen wood as bedding material, 5 mice in each cage, room temperature 22 °C, humidity 40%) under a regular 12-h dark/light cycle. Food and water were available ad libitum.

**GL261 orthotopic model.** 24 mice were orthotopically implanted with the syngeneic cell line GL261-GFP, 5×10^4^ cells in 1 µL PBS into the striatum. With bregma as origin, implantations coordinates were X = 0mm, Y = +2mm, Z = -3mm. Mice were anesthetized with isoflurane (3% for induction and 2% for maintenance) in 100% O_2_. 0.05 mg.kg^-1^ of buprenorphine was subcutaneously administered at the end of the intervention to prolong analgesia.

**MRI.** 14 days after GL261 implementation, anatomical T2-weighted and T1-weighted contrast-enhanced MRI were acquired with a 7T/90mm bore hole MRI scanner (Pharmascan scanner, Bruker). A Gadolinium-based contrast agent (Dotarem^®^, 1nm diameter, 100 µL by animal) was intravenously injected via a catheter. T1-weighted images were then acquired (MSME sequence, TE/TR = 8/340 ms, matrix = 256 × 256 × 64, resolution = 0.15 × 0.15 × 0.60 mm^3^, 10 averages, acquisitions time = 6 min). T2-weigthed images were acquired through a RARE sequence (TE/TR = 5/1800 ms, RARE factor = 16, matrix = 256 × 256 × 64, resolution = 0.12 × 0.12 × 0.12 mm^3^).

**Blood-brain barrier disruption.** A focused transducer (active diameter 25 mm, focal depth 20 mm, axial resolution 5 mm, lateral resolution 1 mm, Imasonic) centered at 1.5 MHz was used to disrupt the BBB. The transducer was connected to a single-channel programmable generator (Image Guided Therapy) and mounted on a motorized XYZ-axis stage. and positioned above the mouse head maintained under anesthesia with isoflurane (3% for induction and 1.5% for maintenance) in a 50:50 mixture of air-O_2_. The device was coupled to the mouse skull using a latex balloon (filled with deionized and degassed water) and centrifugated coupling gel. The distance between the transducer and the skull was adjusted by the displacement of the motorized axis (Z) and the filling of the balloon in order to get the center of the of the brain, at the focal distance (*i.e.*, 20 mm). SonoVue® microbubbles (Bracco) were intravenously administrated in the tail vein via a bolus (50 µL) before the beginning of the FUS or sham sessions. The FUS sequence was similar to the one described in Felix et *al.* and already validated for efficient and safe BBB disruption on healthy mice 28,35. Reversibility of the BBB opening within 24 h after FUS was confirmed in mice using [^18^F]2-fluoro-2-deoxy-sorbitol, a PET marker of BBB integrity (Figure S2). Briefly, the FUS sequence is composed of quasi-continuous ultrasonic waves transmitted with duty cycle of 69% at a peak negative pressure of 420 kPa (considering a transmission through mouse's skull of 80% at 1.5 MHz). A raster scan (XY-axis) of 6 mm x 6 mm was synchronized to the generator output to induce a whole brain BBB opening (Scheme S1). The sequence of 5.1s was repeated 25 times for a total exposure of 126.75 s.

**microPET/CT imaging.** On day 15 post GL261-GFP implementation, a 60-min dynamic PET scan was performed concurrently with radioligand injections of ^89^Zr-DFO-C4 (3.5±0.3 MBq, 4.97 MBq/nmol, n = 8) or ^89^Zr-DFO-C4^Fc-*MUT*^ (3.5 ± 0.5 MBq, 9.89 MBq/nmol, n = 16) under camera after the FUS protocol. The radioligand injection was performed quickly after the end (1.7 ± 0.2 min) of the FUS BBB opening protocol. 20-min static PET scans were subsequently acquired at selected times post-injection (5 h, 22 h, 46 h, 70 h, and 7 days). A sham workflow (without emission of ultrasound waves) was applied on 6 tumor-bearing animals before the injection of ^89^Zr-DFO-C4^Fc-*MUT*^ and imaged accordingly.

PET emission scans were performed using an Inveon microPET scanner and an Inveon microPET/CT scanner (Siemens). After each PET scan, a transmission scan or a CT scan were performed for photon attenuation correction. PET images were reconstructed with the Inveon Acquisition Workspace software (2.1) using a three-dimensional ordinary Poisson ordered-subset expectation maximization followed by a maximum a posteriori algorithm (OP-OSEM3D-MAP). Normalization, as well as corrections for dead-time, scatter, decay and attenuation, were applied to all PET data.

Dynamic PET acquisitions were reconstructed in 24 frames averaging signal on the period from 0.5 to 5 min resulting in a sequence of images of 3 × 30, 5 × 60, 5 × 120, 3 × 180, 3 × 240, 4 × 300, and 1 × 150 s.

Image analysis was performed with the PMOD software (v3.9). A volume of interest (VOI) was defined in the left cardiac ventricle to obtain blood radioligand concentration. MRI and brain PET acquisitions were all repositioned with the T2w MRI as reference. VOI were defined in selected brain areas, defining the T1w contrast-enhanced volume, the PET contrast-enhanced volume, and the contralateral hemisphere. Concentrations in VOI are expressed as percentage of injected dose (%ID/cc = activity (Bq/cc) / injected dose (Bq)) and time activity curves (TACs) were extracted (Table S1-4).

**Immunofluorescence.** After the last imaging session, mice were sacrificed, and their brains were collected, immersed in isopentane, and frozen in liquid nitrogen. A set of fixed frozen brain sections (10 µm) were incubated with a rat anti-mouse PD-L1 primary antibody (1:500, clone 10F.9G2, Biolegend). The slides were then incubated with an AF546-conjugated donkey anti-rat secondary antibody (1:1000, Jackson Laboratories). Adjacent brain sections were incubated with an AF546-conjugated goat anti-human secondary antibody (1:1000, Jackson Laboratories) to stain the injected C4. Adjacent slides were used for hematoxylin/eosin (H&E) staining. Slides were fixed in neutral buffer formalin 10%, then stained with Harris hematoxylin (Sigma) and Eosin Y (Sigma) according to previously reported protocol 36. Immunofluorescent and H&E-stained sections were scanned with a 20x objective using an AxiObserver Z1 microscope (Zeiss).

**Blood pharmacokinetics.** Plasmatic concentrations were calculated from image-derived blood activity concentration drawn in the left cardiac ventricle. Considering that antibodies are restricted to the serum, a blood-to-plasma concentration ratio of 0.55 was used. A bicompartmental model with a first-order elimination function was individually fitted using Phoenix WinNonlin (v.8.3.1, Certara®). Parameters and equations of the model are detailed in supplemental materials. Parameters of the plasma kinetic were intra-individually fixed to apply the same input function to all brain VOI.

**Brain kinetics.** Brain kinetic parameters were obtained by fitting regional TACs to either a modified 1-tissue compartment model (m1TCM) or a modified 2-tissue compartment model (m2TCM) in Phoenix WinNonlin (v.8.3.1, Certara®). These two models were modified to match the transient character of FUS-induced BBB disruption. Parameters and equations of the model are detailed in SI App. Goodness-to-fit of models was determined by generating the Akaike Information Criterion, plots of residuals over time, and plots of individual prediction versus observed concentration (Figure S3-6). The influence of C4 format on kinetic parameters estimations was assessed with a two-factor ANOVA followed by pairwise comparison of mean with Bonferroni's p-value adjustment in R v.4.0.2. Significance was set to 95%.

## Results

**Figure 1:**
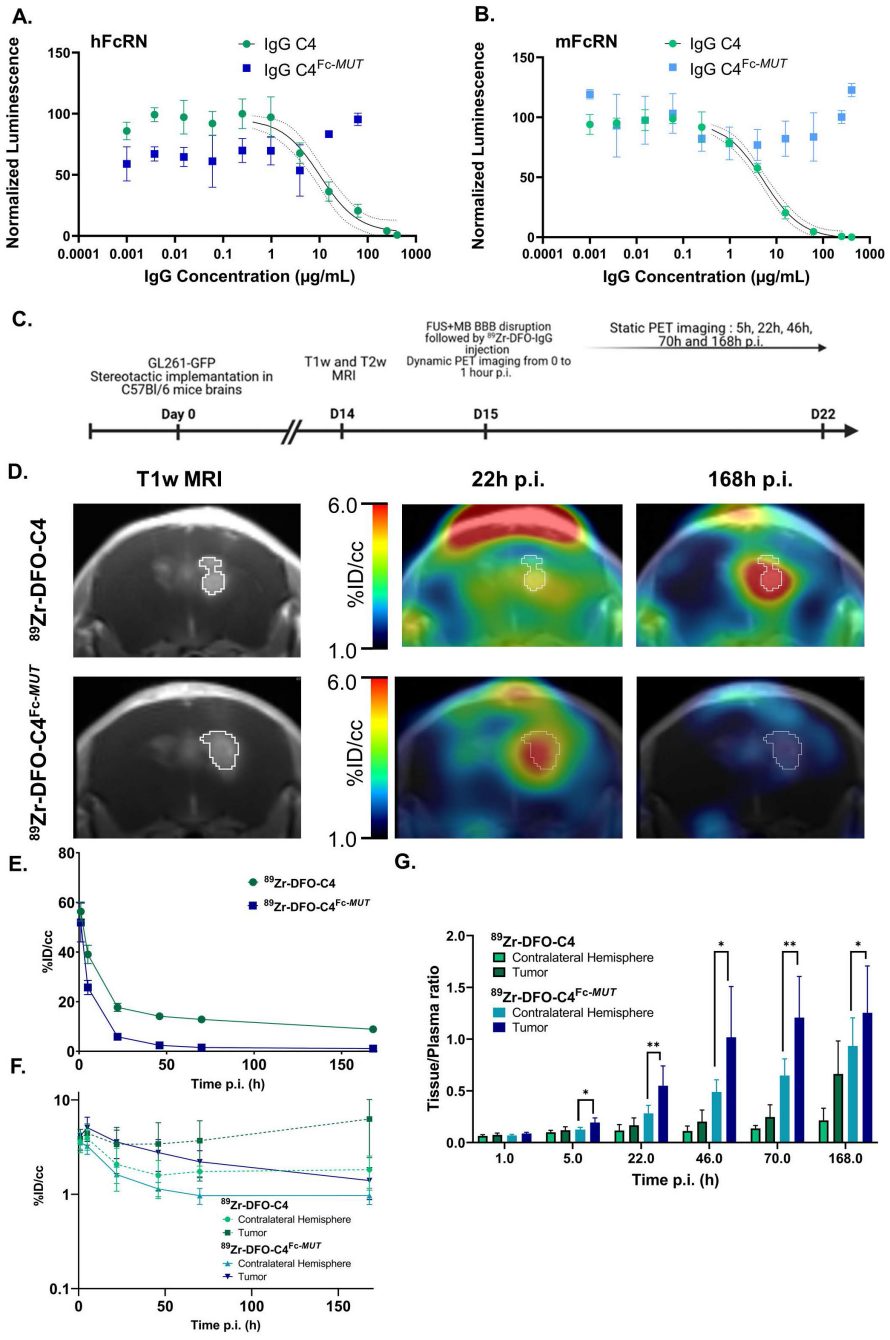
***In vitro* and *in vivo* characterization of the C4 formats for brain PET PD-L1 imaging.** (A, B) Dose-dependent inhibition curves of both C4 formats with human and murine FcRn. Data represent the mean ± SD of triplicate readings. (C) Timeline of the MRI and PET imaging protocol. (D) Representative brain PET- T1w MRI images of GL261-GFP bearing C57Bl/6 mice injected with ^89^Zr-DFO-C4 (n = 6) or ^89^Zr-DFO-C4^Fc-*MUT*^ (n = 8) at 22h and 168h p.i. Time activity curves in plasma (E) and in the brain (F) of the two C4 formats. TACs in the tumor and contralateral hemisphere are differentiated. (F) Tissue to plasma ratio of the two C4 radioligands in tumor and contralateral hemisphere.

**Characterization of the native and the mutant C4.** First, we sought to verify that introducing the H310A and H435Q mutations in the Fc domain of the C4 anti-PD-L1 antibody (C4^Fc-*MUT*^) led to significant differences in affinities for the FcRn. Affinities for the FcRn of both IgG were measured by a competition assay. Incubation with the native C4 led to a concentration-dependent decrease in bioluminescent signal signing the interaction with human (IC_50_ = 10.2 µg/mL) and murine FcRn (IC_50_ = 5.2 µg/mL). No signal inhibition was observed with the C4^ Fc-*MUT*^, which confirmed the abolition of interaction with the FcRn induced by the mutation (Figure 1A-B).

**Lifting Fc/FcRn interaction for brain PET imaging.** We evaluated the potency of anti-PD-L1 IgGs with low affinity for the FcRn receptor for brain PET imaging by comparing the kinetics of ^89^Zr-DFO-C4 and ^89^Zr-DFO-C4^Fc-*MUT*^ in an orthotopic syngeneic mouse model of GBM. Post-contrast T1-weighted images depicted comparable tumor growth between the two mice groups (Figure S7).

As expected, the plasma clearance of ^89^Zr-DFO-C4^Fc-*MUT*^ was faster compared with ^89^Zr-DFO-C4 (Figure 1E). A bicompartmental model with a first-order elimination function was individually fitted to plasma kinetics. Parameters' estimations of the two C4 formats plasmatic kinetic are reported in Table 1. ^89^Zr-DFO-C4^Fc-*MUT*^ is cleared from the central compartment promptly compared with ^89^Zr-DFO-C4, therefore associated with a shorter mean terminal half-life of 89.7 h versus 176.4 h for ^89^Zr-DFO-C4 (p-value = 0.009). ^89^Zr-DFO-C4^Fc-*MUT*^ peripheral distribution volume is twice as high as ^89^Zr-DFO-C4, and its transfer rate from peripheral to the central compartment (k_21_) is significantly lower than IgG C4's one (0.020 ± 0.009 h^-1^ versus 0.055 ± 0.032 h^-1^). Those differences in kinetics are consistent with higher retention of the low FcRn affinity antibody in the liver and the spleen (Figure S8).

FUS enabled the entry into the brain of both radiolabeled antibodies. At 1h post-injection, ^89^Zr-DFO-C4 and ^89^Zr-DFO-C4^Fc-*MUT*^ mean concentrations in the brain reached 3.64 ± 0.73 %ID/cc and 3.75 ± 0.41 %ID/cc, respectively. Reflecting its blood kinetic, the ^89^Zr-DFO-C4^Fc-*MUT*^ concentrations in the contralateral hemisphere decreased at a higher rate than the concentrations of ^89^Zr-DFO-C4 (Figure 1F). The tumor distribution of the two C4 formats differed heavily over time. After reaching the maximal concentration of 5.1 ± 1.5 %ID/cc, ^89^Zr-DFO-C4^Fc-*MUT*^ concentration in the GBM tumor decreased at a slower pace than in the contralateral hemisphere. It resulted in a significant difference of tissue to plasma ratio between the tumor and the contralateral hemisphere from 5h p.i. (Figure 1F). Tumoral uptake of ^89^Zr-DFO-C4, on the contrary, increased from 48h post-injection to reach a maximal uptake 7 days post-injection. Over time, those differences in brain distribution led to the highest PET contrast in the tumor observed at 22h post-injection for ^89^Zr-DFO-C4^Fc-*MUT*^ versus 7 days post-injection for the ^89^Zr-DFO-C4.

**Figure 2:**
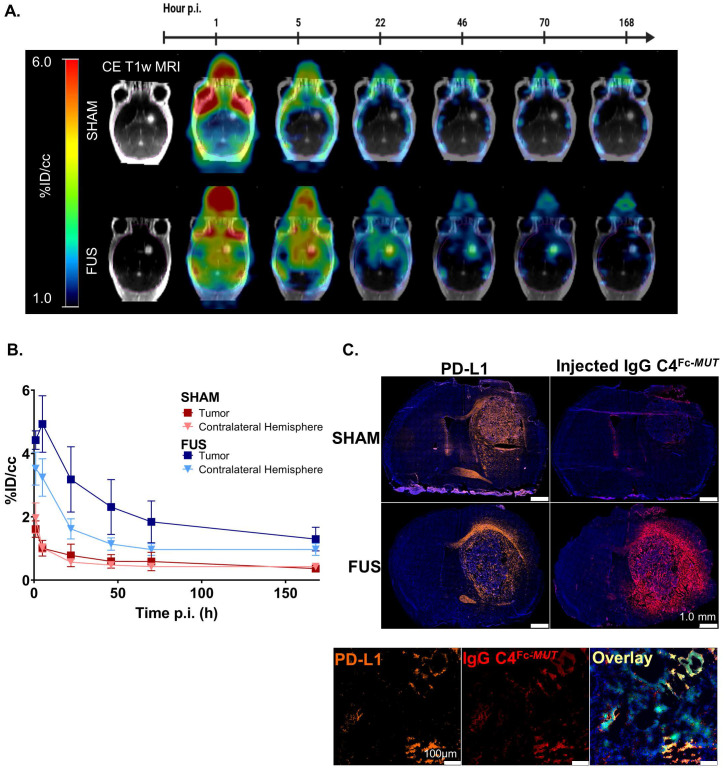
** Brain kinetic of ^89^Zr-DFO-C4^Fc-*MUT*^ after FUS-induced BBB permeabilization.** (A) Representative PET images overlaid on post-contrast T1-weighted MRI from the same mouse at different time points (1h, 5h, 22h, 46h, 70h and 168h after injection) for the sham (*top*) and the FUS (*bottom*) groups. (B) Time activity curves of the ^89^Zr-DFO-C4^Fc-*MUT*^ in the tumor enhanced on T1-weighted MRI and in the contralateral hemisphere in the sham (*red*, n = 5) and the FUS (*blue,* n = 8) groups. All data are represented as mean ± SD. (C) Immunofluorescence staining of brain sections of C4^Fc-*MUT*^ injected mice. Adjacent 10µm cryo-sections were stained with either a rat anti-mouse-PD-L1 IgG and an AF546-goat-anti-rat IgG (orange) or AF546-goat-anti-human IgG (red) to detect the injected antibody. Immunofluorescence signal is overlaid on DAPI images (blue). Magnification of a cryo-section stained with rat anti-mouse-PD-L1 IgG and an AF546-goat-anti-rat IgG (orange) or AF546-goat-anti-human IgG (red) to detect the injected antibody. Immunofluorescence signal is overlaid on DAPI images (blue), GL261 cells express GFP (green). Supplementary immunofluorescence staining are shown in *SI Appendix* (Figure S8).

**FUS-aided immunoPET imaging of glioblastoma.** To validate the benefits of applying FUS to target PD-L1 with ^89^Zr-DFO-C4^Fc-*MUT*^, immunoPET imaging was performed in a GBM model with (FUS group) and without (sham group) FUS-induced BBB permeabilization before the injection. A similar imaging protocol to the one described earlier was applied and we verified that tumor growth was similar between the sham and the FUS group on post-contrast T1-weighted MRI (Figure S7). In addition to the brain, FUS protocol had a significant impact on the spleen uptake of the ^89^Zr-DFO-C4^Fc-*MUT*^ (Figure S8). As expected, BBB permeabilization by FUS significantly enhanced the brain uptake of ^89^Zr-DFO-C4^Fc-*MUT*^ (Figure 2A). One-hour post-injection, brain uptake of ^89^Zr-DFO-C4^Fc-*MUT*^ reached 3.75±0.41%ID/cc with FUS versus 1.92±0.45%ID/cc without FUS. The difference between the two groups remained significant up to 168h post-injection, even in the contralateral hemisphere (Figure 2B). Immunostaining with a secondary antibody targeting the ^89^Zr-DFO-C4^Fc-*MUT*^ confirmed the specificity of the signal observed (Figure 2C). The injected antibody was only detectable in the tumor when FUS was applied, even though PD-L1 was expressed. No evidence of brain damage was observed into the brain after FUS protocol on hematoxylin/eosin staining (Figure S9).

**Figure 3:**
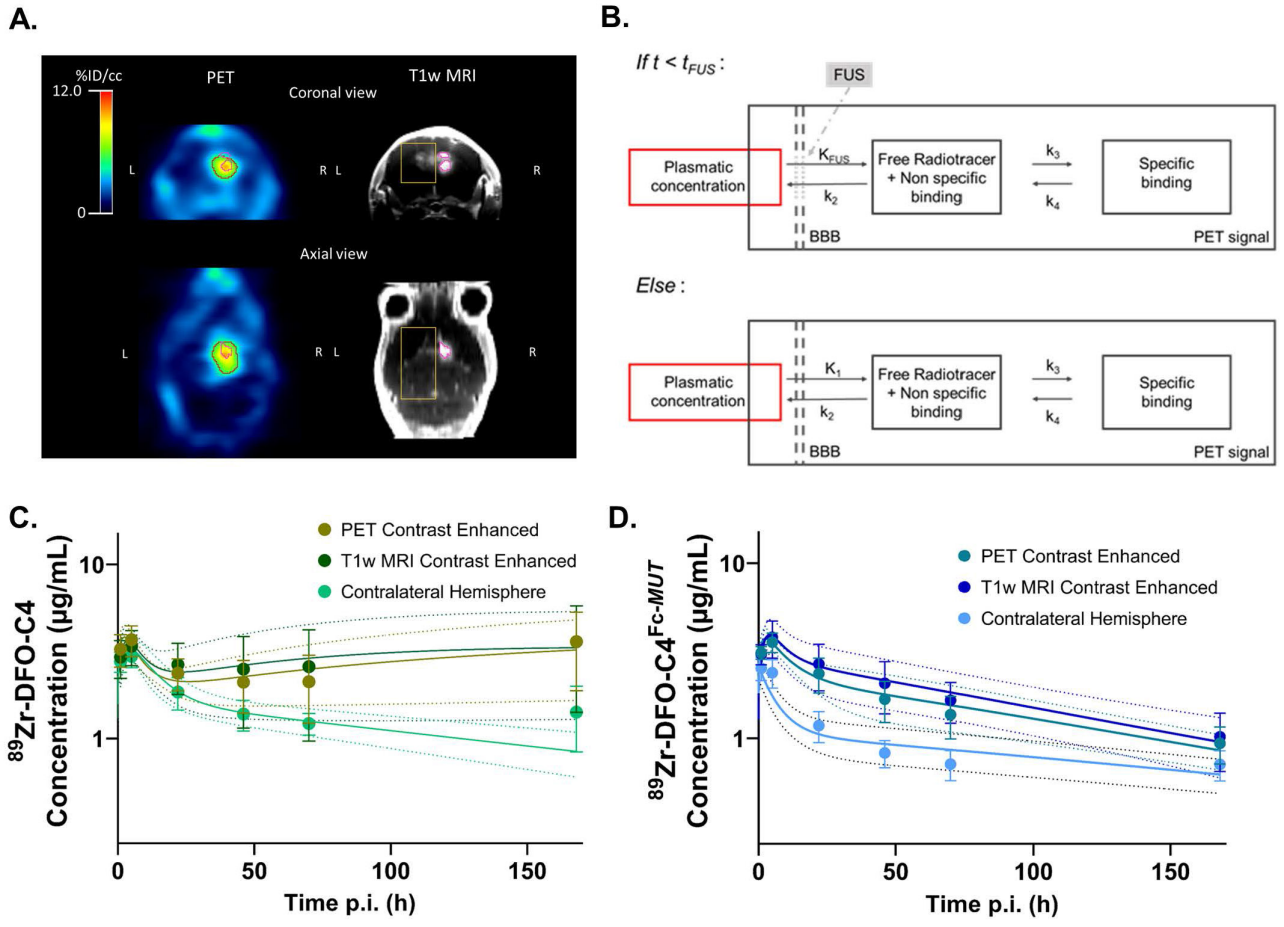
** Brain kinetic modeling to elucidate FcRn's role at the BBB and the impact of FUS-induced BBB disruption on antibodies delivery.** (A) Representative example of VOI identifying the contralateral hemisphere (*yellow*), the tumor enhanced on post-contrast T1-weighted MRI (*pink*) and the tumor enhanced on PET images (*red*). (B) Structure of the 2-tissue compartment model applied on tumoral VOI. Observed (*markers*) and predicted (*solid lines*) brain concentration of ^89^Zr-DFO-C4 (n = 4) (C) and ^89^Zr-DFO-C4^Fc-*MUT*^ (n = 7) (D) according to the different VOI. All data are represented as mean ± SD. *K_FUS_ and K1: perfusion dependent entry constant from plasma to free/non-specifically bound compartment. t_FUS_: time of significant closure of the BBB. k2: transfer constant from free/non-specifically bound compartment to plasma. k3 and k4: transfer constant between free/non-specifically bound compartment and specifically bound compartment. FUS +MB: Focused ultrasound on microbubbles.*

**Kinetic modeling of ^89^Zr-labeled C4 brain PET data.** To further decipher the role of FcRn at the BBB and characterize the impact of FUS on the entry of antibodies in the brain, compartmental modelling was performed (Figure 3A). As no specific binding is expected in the contralateral hemisphere, a 1-Tissue compartment model was chosen. For the tumoral volume, a 2-Tissue compartment model was fitted (Figure 3B). Mean predicted and observed data of ^89^Zr-DFO-C4 and ^89^Zr-DFO-C4^Fc-*MUT*^ versus time in each brain volume are displayed in figures 3C and 3D, respectively.

Our structural model introduced two supplemental parameters, t_FUS_ which describes the time for the BBB to recover integrity after FUS, and K_FUS_, which describes the perfusion-dependent transfer rate of IgG across the permeabilized BBB (Figure 3B). K_FUS_ and t_FUS_ estimates did not differ between the two ^89^Zr-DFO-C4 formats or across brain regions (Table 2). FUS enabled the entry of the two antibodies at a mean rate of 0.163±0.071 mL/h/g of tissue for 12.1±4.6 minutes after the end of the FUS protocol.

The impaired interaction of ^89^Zr-DFO-C4^Fc-*MUT*^ with FcRn significantly decreased the efflux rate constant (*k*_2_) from the healthy brain tissue to plasma (0.015±0.027 h^-1^) compared with ^89^Zr-DFO-C4 (0.300±0.218 h^-1^, p-value = 0.002). It also impacted the influx rate constant (*K*_1_) post-FUS, with a 10-fold decrease in the rate constant for transfer from plasma to tissue (Table 2). However, in the tumoral volumes, there was no significant difference between both ^89^Zr-DFO-C4 formats' *K*_1_ and *k*_2_ estimates.

## Discussion

Focused ultrasound is an emerging technology that could change the paradigms of glioblastoma treatment. If associated with immunotherapy, there is a need for a companion methodology to select patients that will respond to such therapy. Improving the kinetic of antibodies in brain tumors is essential for therapy and imaging purposes. The difficulty in addressing personalized therapy for GBM prompted us to propose a theranostic approach based on quantifying PD-L1 expression in brain lesions.

Antibody design with low FcRn affinity has been described as a promising strategy to overcome some pharmacokinetic limitations of immunoPET imaging. Bouleau *et al.* demonstrated that using an H310A/H435Q mutant-IgG offers more advantages for immunoPET imaging than using smaller objects, which mainly aims to decrease the plasma half-life. They obtained significantly higher tumoral uptake of the ^89^Zr-C4^Fc-*MUT*^ compared with the corresponding ^89^Zr-Fab while benefiting from a fairly fast blood clearance resulting in high contrast PET images at only 24h post-injection 17. Although human IgG1 has a stronger affinity for murine FcRn than for human FcRn, which could impact the prediction of a human antibody pharmacokinetic from mice models, the H310A/H435Q mutation has a translational value as it abolishes IgG1 interaction with both the human and the murine FcRn 14,16. Moreover, the importance of FcRn functions on the transcytosis and recycling of IgGs is preserved across species 37.

**Figure 4:**
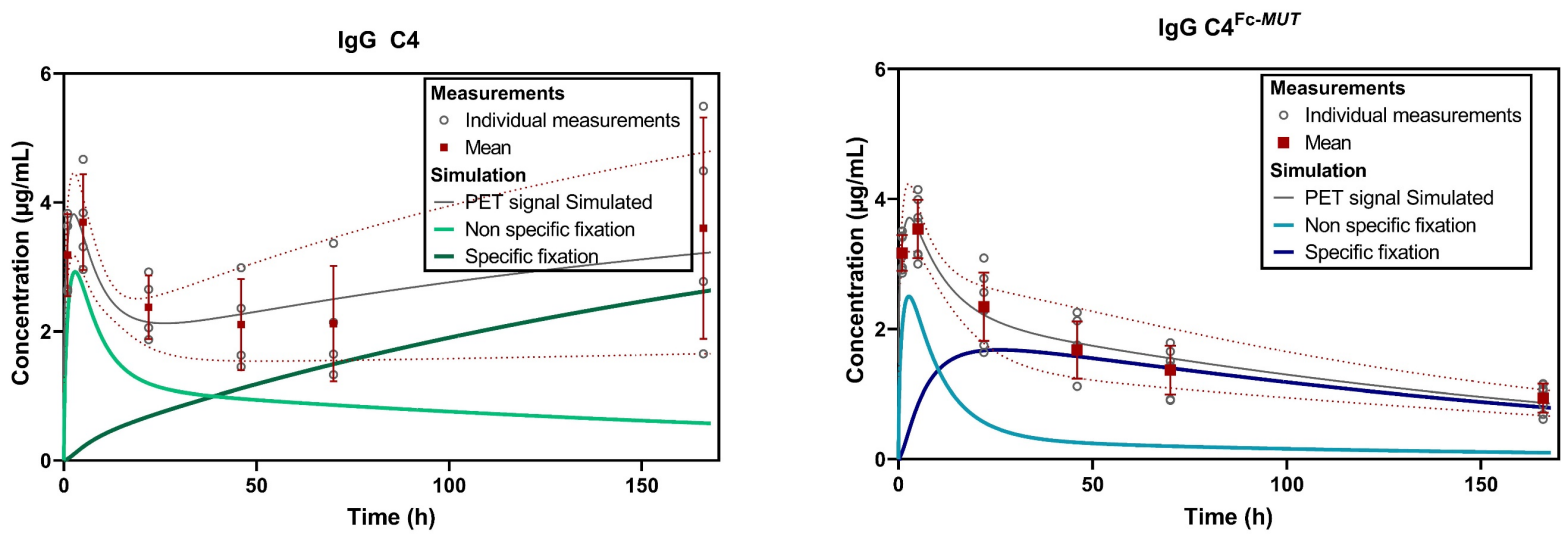
** Simulated concentrations of the two C4 formats in GBM tumor with a preserved BBB integrity on T1w post-contrast MRI.** Concentrations were normalized to an injected dose of 70µg of antibody. The PET signal simulated is the resultant of the sum of the non-specific and specific fixation. Parameters used for the simulation were the mean of individual estimated parameters.

The overall brain exposure of ^89^Zr-DFO-C4^Fc-*MUT*^ was lower than that of ^89^Zr-DFO-C4. This decrease in brain disposition was similarly observed by Chang *et al.*, who compared the brain kinetics of the i.v administered human IgG1 trastuzumab and its (I253A/H310A/H435A) mutant, which is unable to bind FcRn 38,39. The retention of the mutated C4 due to the binding to PD-L1, enabled by FUS, did not compensate for the reduced brain disposition compared with the ^89^Zr-DFO-C4. The dose-normalized simulation of both C4 formats kinetic in the tumor considering a single intravenous injection and FUS-induced BBB disruption shows that tumor IgG exposure overtime was higher for the ^89^Zr-DFO-C4 (AUC_0-168h, C4_ = 433 μg*h/mL) compared to the mutated IgG (AUC_0-168h, C4(Fc-*MUT*)_ =247 μg*h/mL) (Figure 4). Therefore, using IgG with a modified Fc to reduce their affinity for FcRn does not appear advantageous for therapeutic purposes. These results are the consequence of the significantly reduced plasma half-life of ^89^Zr-DFO-C4^Fc-*MUT*^ due to the abolition of FcRn-mediated transcytosis and recycling. In fact, the high brain exposure of ^89^Zr-DFO-C4 might result from the BBB disruption associated with GBM at an advanced stage at 21 days post-implementation, in addition to the long plasma half-life of IgGs 40,41. ^89^Zr-DFO-C4^Fc-*MUT*^ foremost benefits from improved kinetic properties for PET imaging of brain tumors expressing PD-L1 as it generates an optimal contrast between the brain tumor and the contralateral hemisphere at 22h p.i. for the ^89^Zr-DFO-C4^Fc-*MUT*^ against 168 h for ^89^Zr-DFO-C4. Kinetic modeling was used to decipher the non-specific and the specific binding contribution to the observed PET signal. It enlightens the potency of ^89^Zr-DFO-C4^Fc-*MUT*^ to measure target engagement with the specific binding being predominant as soon as 12 h post-injection, versus 48 h post-injection for the full IgG, ^89^Zr-DFO-C4 (Figure 4). Moreover, the low disposition of ^89^Zr-DFO-C4^Fc-*MUT*^ in the brain without FUS-induced BBB disruption, associated with its fast plasma clearance, implies that the PET image predominantly reflects PD-L1 expression at the time of injection. The same assumption cannot be made for the ^89^Zr-DFO-C4 which uptake may depend on the growth and evolution of the tumor between the time of injection and optimal time for PET acquisition.

FUS were necessary to image GBM with the ^89^Zr-DFO-C4^Fc-*MUT*^. Although BBB disruption was induced on both brain hemispheres, the binding of ^89^Zr-DFO-C4^Fc-*MUT*^ was mostly observed in the GBM tumor volume, thus confirming the specificity of the PET signal for PD-L1 expression. The maximum concentration in the GBM tumor was attained at 5 h post-injection versus 1h post-injection in the contralateral hemisphere. This delay reflects the time of association of ^89^Zr-DFO-C4^Fc-*MUT*^ liberated in the interstitial fluid of the brain parenchyma to its target, PD-L1. Safety of the FUS protocol used was previously validated by T2w and T2* MRI in addition to histological analysis 28,42,43. Concordantly, no structural impact of FUS was observed on the brain of mice on H&E staining.

Kinetic modeling of the impact of FUS on the brain kinetics of a radiolabeled compound was previously described using a classic 1-tissue compartment model 44. This model implies that the entry rate of the radiotracer is constant throughout the observation period. In this study, we considered a discontinuous entry function to match the transient character of FUS-induced BBB disruption. To this end, we introduced two additional parameters related to the molecule uptake, K_FUS_ and t_FUS_, that correspond respectively to the clearance and to the time of BBB/BTB sealing after the transient disruption with FUS. The similarity of these two parameters in all brain regions where FUS were applied reinforces the hypothesis that they only are related to the dynamics of BBB permeation/recovery rather than the intrinsic kinetic properties of tested antibodies. Considering all brain regions and for both C4 formats, the mean K_FUS_ is 0.163±0.071 mL/h/g of tissue. The same order of magnitude with the uptake clearance calculated for ^89^Zr-Cetuximab passage has been observed by Tran *et al.* (CL_up Cetuximab_= 0.78±0.36 mL/h/g of tissue) 28. The mean t_FUS_ was determined to be 10.4 ± 4.6 min, which is consistent with the theoretical closure half-life of the BBB from Marty *et al.* 45. Their estimation, based on semi-empirical observation with MRI revealed a half-life of 17.8 min after FUS-enhanced delivery of particles with a hydrodynamic diameter similar to a diameter of an antibody (≈10 nm). Additionally, in our case, implementing a discontinuous permeability function improved the model's fit with the observed concentrations in the tumor volume delineated by contrast-enhanced T1w-MRI. In this particular volume, the BBB is already disrupted by the GBM presence, allowing diffusion of gadoteric acid in the tumor. This suggests that FUS transiently induced a stronger disruption of the BTB than the one induced by this particular tumor model. Enhanced BTB permeability induced by FUS remains to be further investigated in other preclinical models. Of note, this was not observed by Brighi *et al.*, who performed FUS-aided immunoPET imaging of a patient-derived glioma model in mice 46.

The robustness of the estimation of K_FUS_ and t_FUS_ allowed the characterization of our two C4 formats brain kinetic to assess the influence of FcRn affinity quantitatively. In the contralateral hemisphere devoid of tumoral tissue, the ^89^Zr-DFO-C4^Fc-*MUT*^ is associated with a lower efflux rate between the brain and plasma. The influx rate constant was also lower when affinity with FcRn is lost. These results suggest that antibody-FcRn interaction controls the mAbs transcytosis across the BBB in both directions. This conclusion is consistent with the slower clearance from the brain of IgG with reduced affinity observed when the IgG is intracranially injected 21,47. In regions associated with GBM tumoral tissues, transfer rate constants of ^89^Zr-DFO-C4^Fc-*MUT*^ were higher than those in the contralateral hemisphere and were similar to that observed with the unmodified ^89^Zr-DFO-C4. The absence of exchange constants difference between the PET contrast-enhanced and the T1w MRI contrast enhanced volume indicates that mAbs tumoral distribution does not relate to antibody-FcRn interaction or BBB integrity before FUS application but mainly on the antigen presence.

Abolishing Fc-FcRn interaction confers improved kinetic properties to ^89^Zr-DFO-C4*^Fc-MUT^* for immunoPET imaging with a better contrast obtained sooner in brain tumoral tissues. FUS-aided BBB/BTB disruption enables quantitative imaging of PD-L1 expression by glioblastoma tumors within the brain and allowed us to study the effect of Fc-FcRn interaction on the brain distribution of antibodies.

This study demonstrates the potency of FUS-aided BBB disruption with the smart design of radiolabeled antibodies to enable quantitative immunoPET imaging of PD-L1 within the brain.

## Supplementary Material

Supplementary figures and tables.

## Figures and Tables

**Table 1:** Plasma pharmacokinetic parameters of the ^89^Zr-DFO-C4 and the ^89^Zr-DFO-C4^Fc-*MUT*^.

		^89^Zr-DFO-C4				^89^Zr-DFO-C4^Fc-*MUT*^				
Parameter		Mean		Sd		Mean		Sd		p-value
V_c_ (mL)		1.5		0.1		1.7		0.3		0.45
k_10_ (h^-1^)		0.014		0.004		0.076		0.011		7.4E-06
k_12_ (h^-1^)		0.119		0.058		0.094		0.047		0.48
k_21_ (h^-1^)		0.055		0.032		0.02		0.009		0.03
V_p_ (mL)		3.4		0.5		7.8		2.7		0.01
Initial t_1/2_ (h)		4.3		1.5		4.2		1.3		0.84
Terminal t_1/2_ (h)		176.4		50		89.7		30.9		0.009
AUC_0→∞_ (µg·h·mL^-1^)		5954		1411		569		108		1.1E-05

Mean parameters estimates and associated standard errors obtained by individually fitting a bicompartmental kinetic to plasmatic concentration of ^89^Zr-DFO-C4 (n = 6) and ^89^Zr-DFO-C4^Fc-*MUT*^(n = 8). p-value shown are results from Student comparison of mean*. V_c_: volume of the central compartment; k_10_: elimination constant from the central comportment; k_12_: transfer constant from the central to the peripheral compartment; k_21_: transfer constant from peripheral to central compartment; Vp: volume of the peripheral compartment; Initial t_1/2_: initial half-life; Terminal t_1/2_: terminal half-life.*

**Table 2:** Mean estimates and standard deviation of parameters of the different models applied to brain VOI data to characterize ^89^Zr-DFO-C4 and ^89^Zr-DFO-C4^Fc-*MUT*^ kinetic.

			^89^Zr-DFO-C4				^89^Zr -DFO-C4^Fc-*MUT*^					
*Brain region*	Parameter		Mean Estimate		Sd		Mean Estimate		Sd		p-value	
*Contralateral*												
	K_FUS_ (mL/h/g of tissue)		0.167		0.156		0.094		0.037		ns	
	t_FUS_ (h)		0.165		0.099		0.215		0.065		ns	
	K_1_ (mL/h/g of tissue)		0.030		0.019		0.003		0.005		*0.005*	
	k_2_ (h^-1^)		0.300		0.218		0.015		0.027		*0.002*	
	vB		0.064		0.006		0.077		0.014		ns	
												
*T1w MRI CE*												
	K_FUS_ (mL/h/g of tissue)		0.179		0.046		0.200		0.087		ns	
	t_FUS_ (h)		0.142		0.077		0.179		0.102		ns	
	K_1_ (mL/h/g of tissue)		0.053		0.021		0.062		0.028		ns	
	k_2_ (h^-1^)		0.566		0.188		0.601		0.308		ns	
	vB		0.052		0.013		0.053		0.009		ns	
	k_3_ (h^-1^)		0.027		0.017		0.111		0.063		*0.031*	
	k_4_ (h^-1^)		0.005		0.006		0.020		0.011		*0.039*	
												
*PET CE (wo T1w MRI CE)*												
	K_FUS_ (mL/h/g of tissue)		0.155		0.049		0.188		0.077		ns	
	t_FUS_ (h)		0.150		0.037		0.161		0.070		ns	
	K_1_ (mL/h/g of tissue)		0.069		0.033		0.051		0.026		ns	
	k_2_ (h^-1^)		0.742		0.393		0.491		0.298		ns	
	vB		0.058		0.010		0.063		0.013		ns	
	k_3_ (h^-1^)		0.017		0.013		0.077		0.047		*0.036*	
	k_4_ (h^-1^)		0.000		0.000		0.015		0.007		*0.003*	
												

K_FUS_ and K1: perfusion dependent entry constant from plasma to free/non-specifically bound compartment. t_FUS_: time of significant closure of the BBB. k_2_: transfer constant from free/non-specifically bound compartment to plasma. k_3_ and k_4_: transfer constant between free/non-specifically bound compartment and specifically bound compartment. vB: fraction of blood in the tissue.

## Abbreviations

BBB: blood-brain-barrier
BTB: blood-tumotal barrier
CNS: central nervous system
FcRn: neonatal Fc receptor
FUS: focused ultrasound
GBM: glioblastoma
HPLC: high-performance liquid chromatography
ICI: immune checkpoint inhibitor
iTLC: immunoglobuline G
MB: microbubble
MRI: magnetic resonance imaging
PD1: programmed cell death protein 1
PD-L1: programmed death ligand 1
PET: positron emission tomography
