# Supplementary material for: Efficient PD-L1 imaging of murine glioblastoma with FUS-aided immunoPET by leveraging FcRn-antibody interaction
Source: Theranostics. 2023 Oct 16;13(15):5584–96. doi: 10.7150/thno.87168 (PMC10614689; doi:10.7150/thno.87168)
Supplement: Supplementary file 1 — Supplementary figures and tables. [file thnov13p5584s1.pdf]

Supplemental Material

SUPPLEMENTAL METHODS

Radiochemical purity. Assessment of radiochemical purity by HPLC after radiolabeling of the C4 ligands with ^{89}Zr.

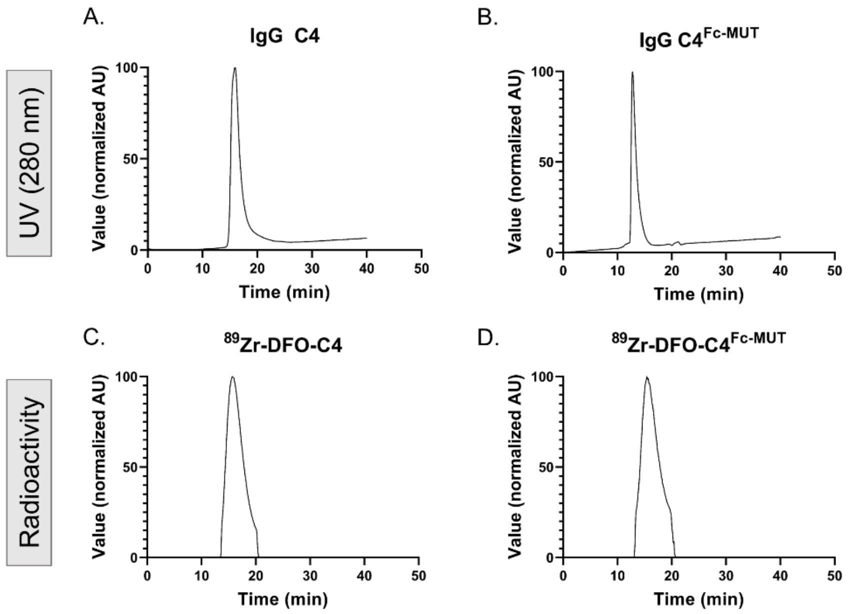

Fig. S1. Representative HPLC chromatograms of purified ^{89}Zr-DFO-C4 (A and C) and ^{89}Zr-DFO-C4$^{Fc\text{-}MUT}$ (B and D) with UV detection at 280 nm (A and B) and radioactivity detection (C and D).AU: arbitrary unit, UV: ultraviolet.

***Ex vivo* biodistribution.** After the last PET acquisition, under deep anesthesia, blood was collected from mice by cardiac puncture in heparinized tubes. Plasma was collected after centrifugation (10 min, 4000 rpm, 4°C). Heart, liver, spleen, kidney, muscle, and bone were collected, weighted and their activity was measured with a Cobra gamma counter (Packard). Background noise and decay correction were applied to the activity measurements. Then injected dose and weight of the organ corrections were performed to obtain data in injected dose per volume of tissue (%ID/cc).

FUS device.

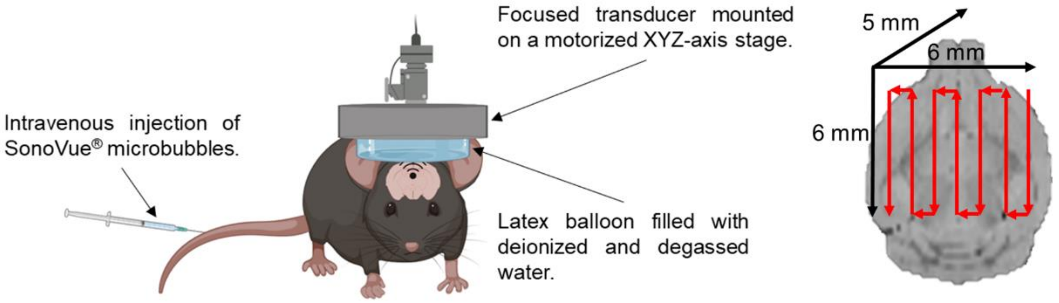

Scheme S1. Scheme of the device used to disrupt mice's blood-brain-barrier and of the transducer trajectory on mice skulls.

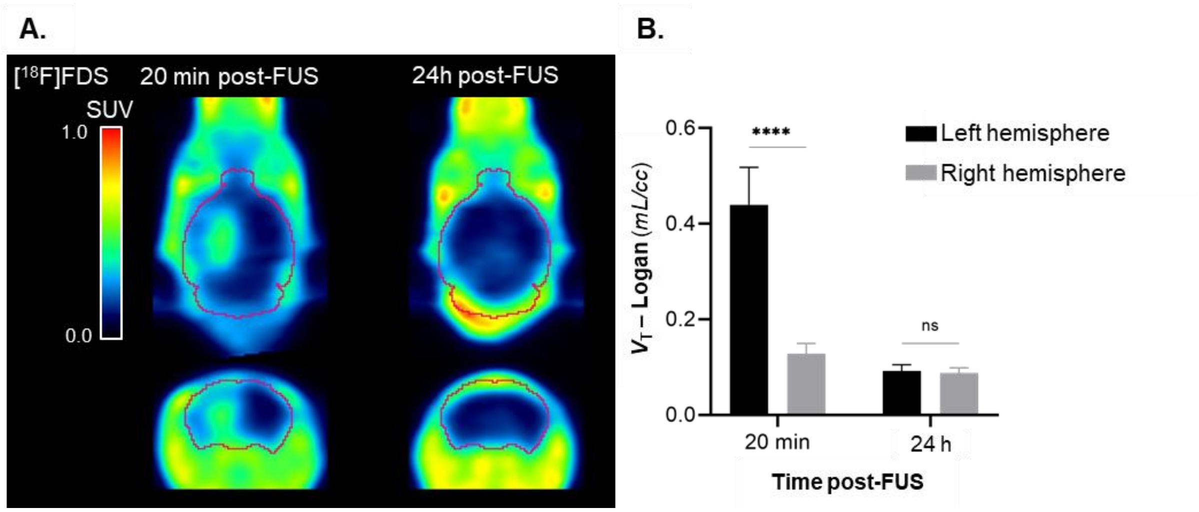

Fig. S2. Reversibility of the blood-brain barrier opening by FUS assessed by [^{18}F]2-fluoro-2-deoxy-sorbitol ([^{18}F]FDS) PET imaging. BBB disruption was induced by FUS on the left hemisphere, and [^{18}F]FDS was intravenously injected immediately after (6.4±0.7 MBq, n = 5) and 24h after (6.1±0.4 MBq, n=5) in the same C57Bl/6 healthy mice. FUS and PET imaging protocols are described by Hugon et al. (A) Standardized uptake value (SUV) normalized brain PET images of [^{18}F]FDS uptake 20 minutes and 24 hours after hemispheric BBB disruption induced by FUS. (B) Total volume of distribution (V_T) was estimated using the Logan graphical analysis. Statistical significance was determined using a t-test with **** p-value < 0.0001. Data are represented as mean ± SD.

Blood kinetic equations. A two-compartment model with intravenous bolus input and first-order elimination was used to fit the plasma time-activity curves of mice injected with the C4 radioligands. Equations of the model are:

$$C_{plasma}(t) = \frac{Dose}{V_c} * \left(\frac{k_{21} - \alpha}{\beta - \alpha} e^{-\alpha \times t} + \frac{k_{21} - \beta}{\alpha - \beta} e^{-\beta \times t}\right)$$

And,

$$\mathrm{V_p} = \frac{k_{12} \times V_c}{k_{21}}$$

With V_c: volume of the central compartment; k10: elimination constant from the central comportment; k12: transfer constant from the central to the peripheral compartment; k21: transfer constant from peripheral to central compartment; V_p: volume of the peripheral compartment.

m1TCM and m2TCM equations. A discontinuous entry function was introduced to fit the IgG brain kinetic in area where FUS were applied. m1TCM equations are:

$$\begin{cases} If\ t \leq T_{FUS}: \\ \frac{dC_{Tissue}}{dt} = K_{FUS} \times C_{Plasma}(t) - k2 \times C_{Tissue}(t) \\ Else: \frac{dC_{Tissue}}{dt} = K1 \times C_{Plasma}(t) - k2 \times C_{Tissue}(t) \end{cases}$$

$$C_{VOI}(t) = (1 - vB) \times C_{Tissue} + vB \times C_{Blood}(t)$$

With C_{Tissue} the concentration of ^{89}Zr-DFO-IgG in the considered tissue, K1 (mL/h/g of tissue) a perfusion-dependent entry constant, k2 (h^{-1}) a transfer constant, T_{FUS} (h) the time of closure of the BBB and K_{FUS} (mL/h/g of tissue) the perfusion-dependent transfer constant when the BBB is disrupted. vB (%) is blood volume fraction.

Similarly, m2TCM equations are:

$$\begin{cases} If\ t \leq T_{FUS}: \\ \frac{dC_{NS}}{dt} = K_{FUS} \times C_{Plasma}(t) - (k2 + k3) \times C_{NS}(t) + k4 \times C_S(t) \\ Else: \frac{dC_{NS}}{dt} = K1 \times C_{Plasma}(t) - (k2 + k3) \times C_{NS}(t) + k4 \times C_S(t) \end{cases}$$

$$\frac{dC_S}{dt} = k3 \times C_{NS}(t) - k4 \times C_S(t)$$

$$C_{VOI}(t) = (1 - vB) \times \left(C_{NS}(t) + C_S(t)\right) + vB \times C_{Blood}(t)$$

With C_{NS} the concentration of ^{89}Zr-DFO-IgG non-specifically bind in the tissue and C_S the concentration of ^{89}Zr-DFO-IgG specifically bind in the tissue. k3 and k4 are transfer constant (h^{-1}).

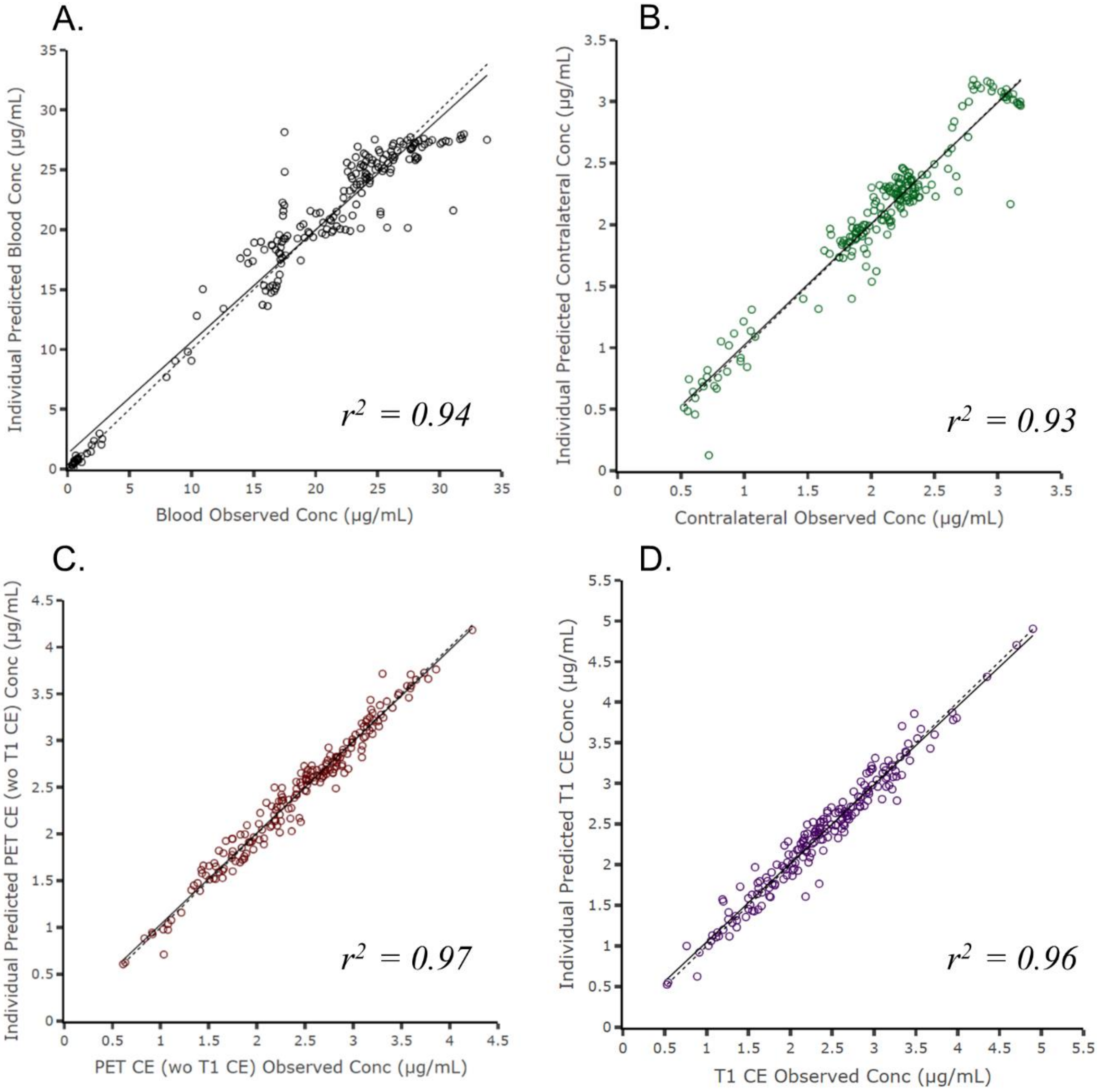

Fig. S3. Scatter plot and regression lines (solid) of IgG C4 observed and predicted concentrations in blood (A), and brain regions: contralateral hemisphere (B), PET CE (C), T1w MRI CE (D). The dotted lines represent the line of identity.

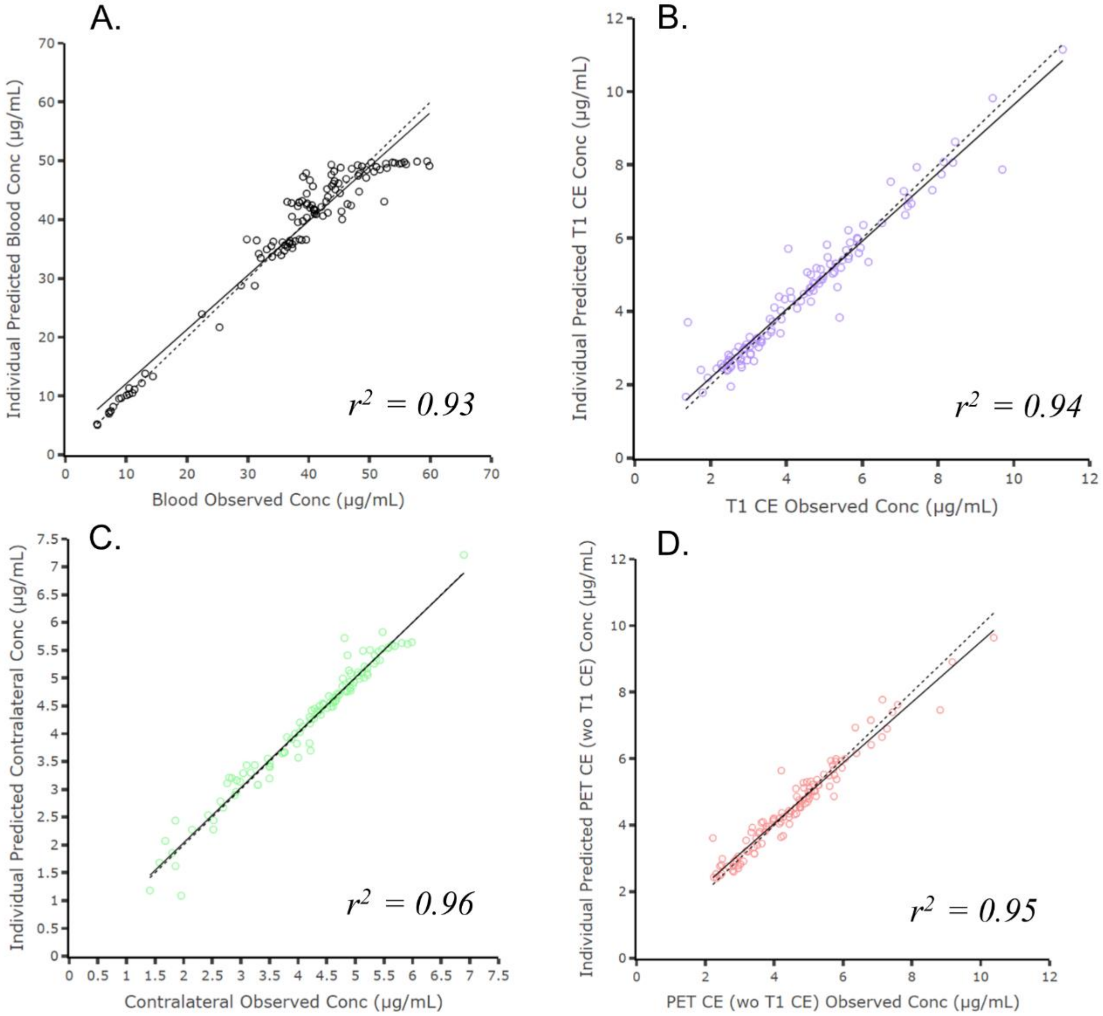

Fig. S4. Scatter plot and regression lines (solid) of IgG $C4^{Fc\text{-}MUT}$ observed and predicted concentrations in blood (A), and brain regions: contralateral hemisphere (B), PET CE (C), T1w MRI CE (D). The dotted lines represent the line of identity.

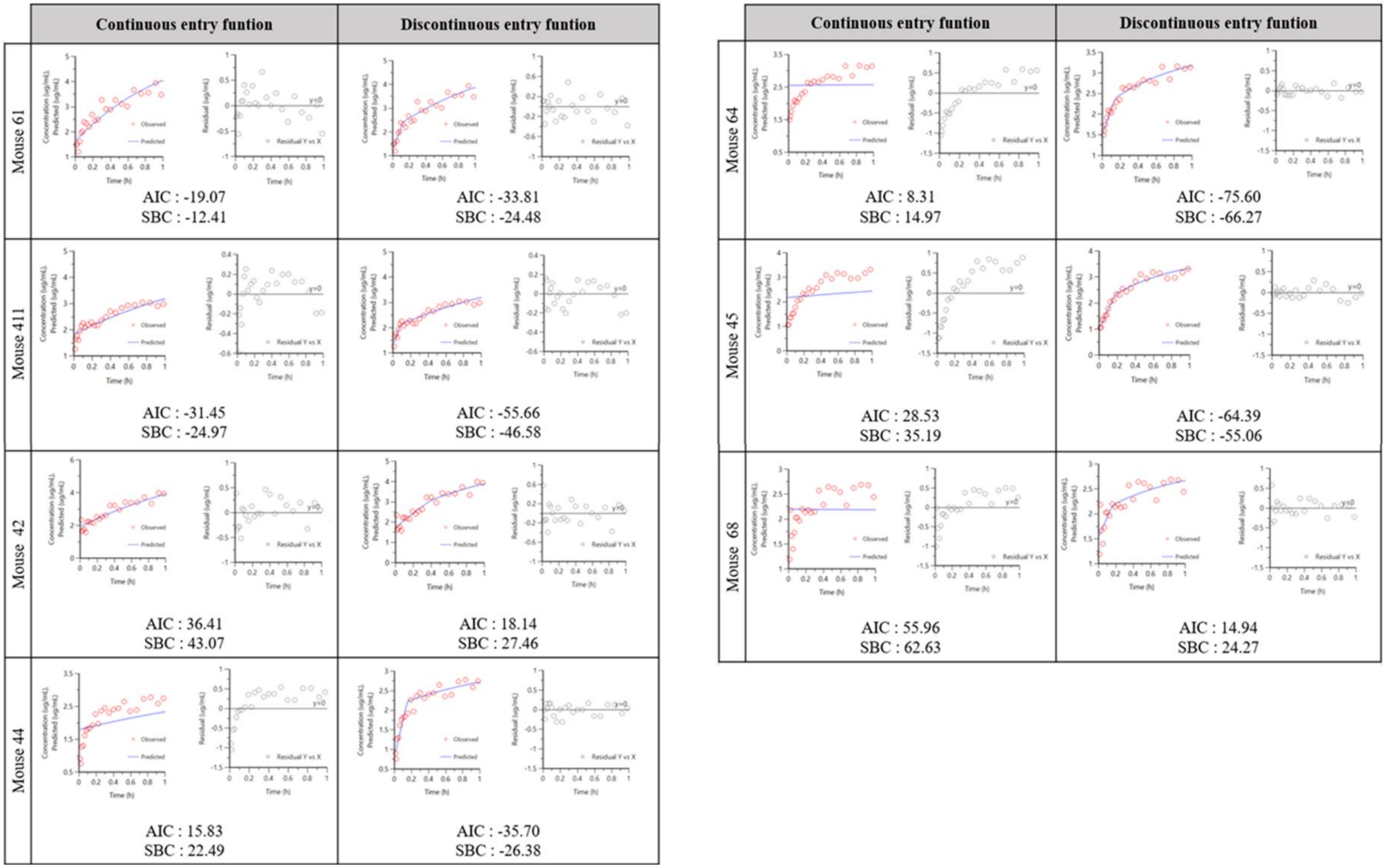

Fig. S5 Elements to evaluate accuracy of the fit models according to the entry function applied in animal injected with IgG C4$^{Fc\text{-}MUT}$ in the T1w MRI contrast enhanced brain region. For each individual and model (left to right): graphic of predicted (blue line) and observed (red circle) concentrations during the first hour post-injection; graphic of associated residuals (grey circle) according to the time post-injection. AIC: Akaike criterion; SBC: Schwarz Bayesian criterion.

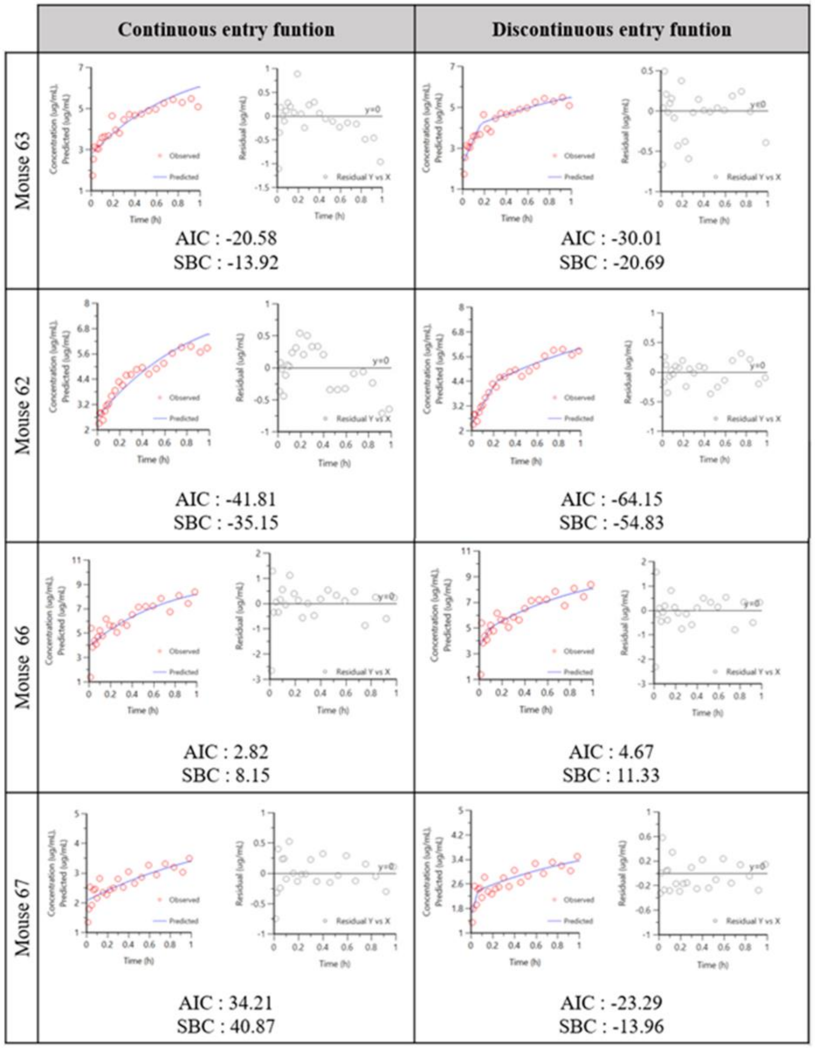

Fig. S6 Elements to evaluate accuracy of the fit models according to the entry function applied in animal injected with IgG C4. For each individual and model (left to right): graphic of predicted (blue line) and observed (red circle) concentrations during the first hour post-injection; graphic of associated residuals (grey circle) according to the time post-injection. AIC: Akaike criterion; SBC: Schwarz Bayesian criterion.

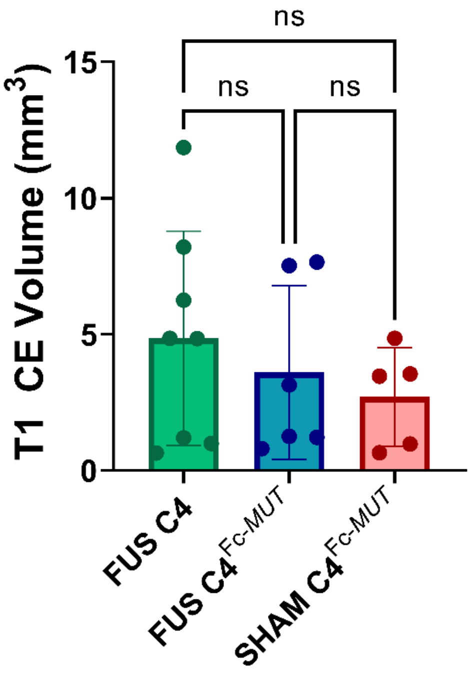

Fig. S7. Comparison of the contrast enhanced tumor volumes on the T1-weighted MRI of the sham and FUS groups injected with C4 (n=8) or C4$^{Fc\text{-}MUT}$ (SHAM: n=5, FUS: n=7) antibodies.

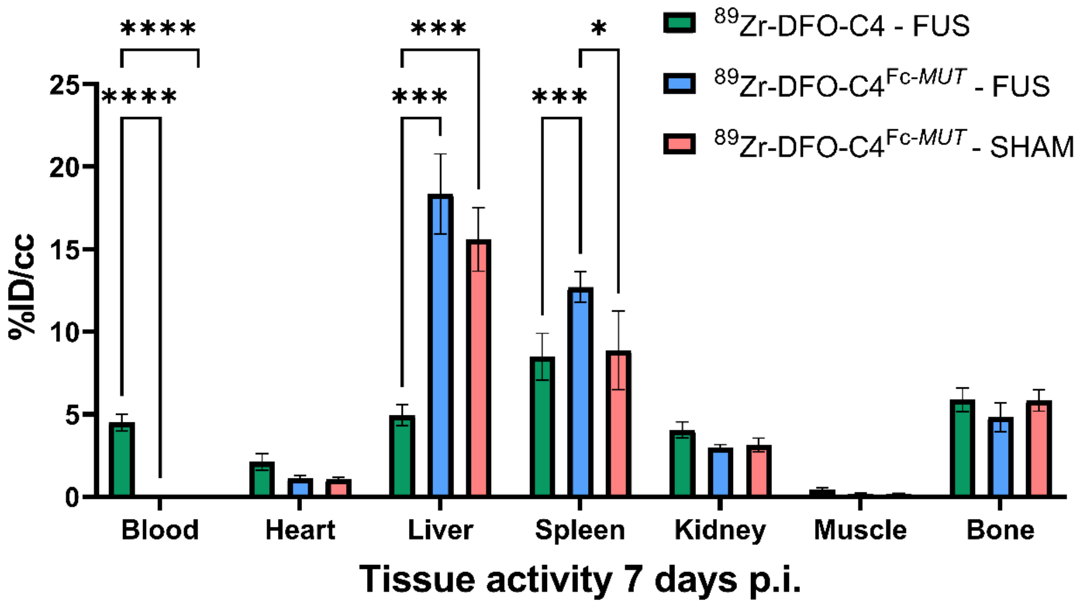

Fig. S8. *Ex vivo* biodistribution of ^{89}Zr-DFO-C4 (n=8) or ^{89}Zr-DFO-C4$^{Fc\text{-}MUT}$ (SHAM: n=5, FUS: n=5), antibodies in the FUS and the sham groups.

A.

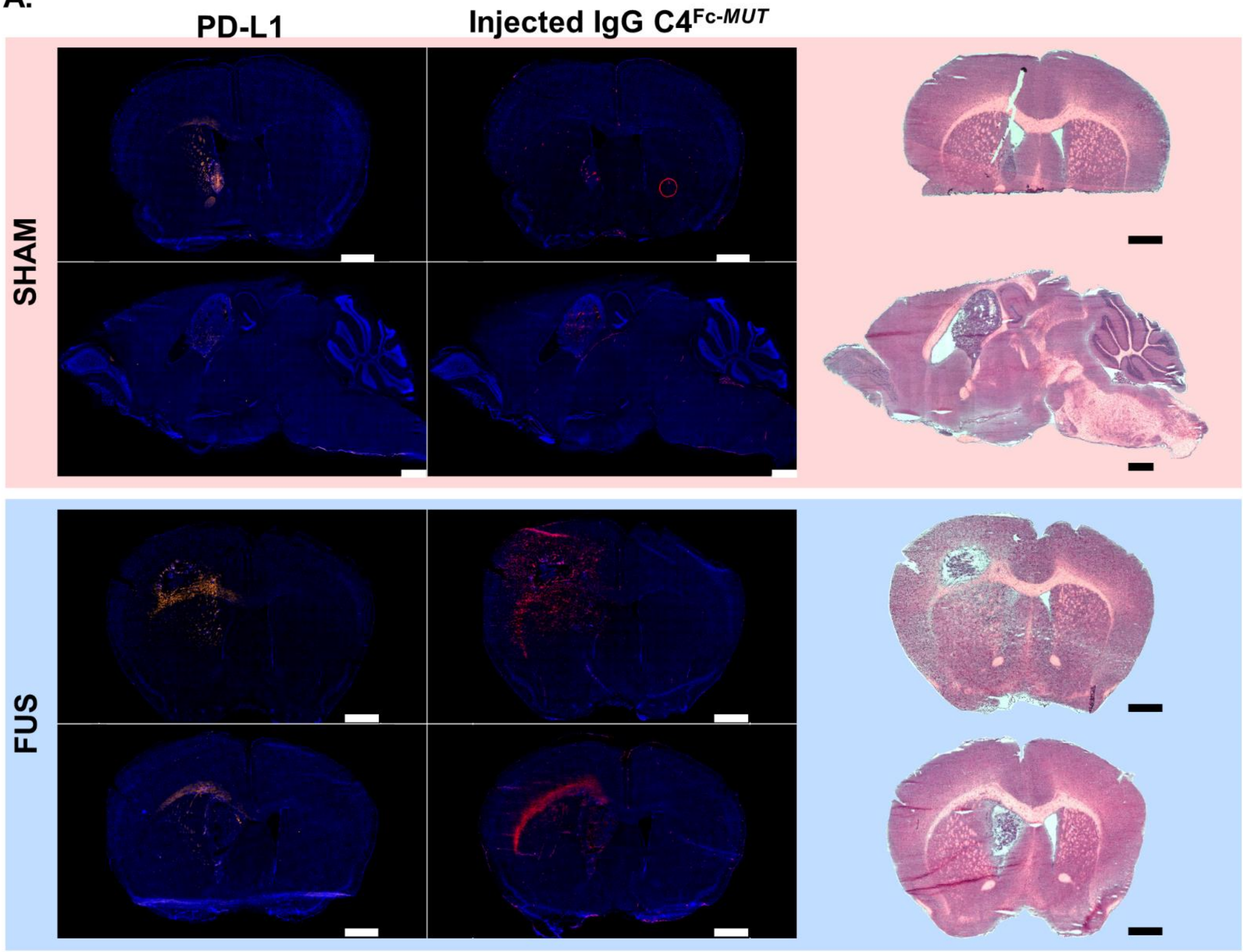

B.

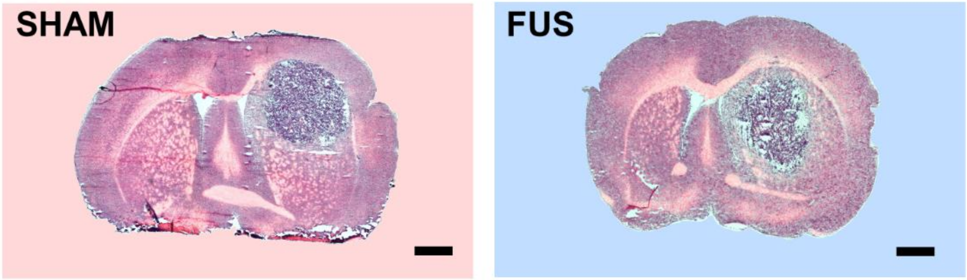

Fig. S9. Immunofluorescence and hematoxylin/eosin staining of brain sections of C4$^{Fc\text{-}MUT}$ injected mice. (A) Adjacent 10µm cryo-sections were stained with either a rat anti-mouse-PD-L1 IgG and an AF546-goat-anti-rat IgG (orange) or AF546-goat-anti-human IgG (red) to detect the injected antibody. The immunofluorescence signal is overlaid on DAPI images (blue). Hematoxylin/eosin staining of adjacent cryo-sections. (B) Hematoxylin/eosin staining of adjacent sections of the ones in Figure 2C of the main manuscript. Scale bar = 1.0 mm.

Table S1. Individual plasma concentration of ^{89}Zr-DFO-C4 and ^{89}Zr-DFO-C4$^{Fc\text{-}MUT}$ from mice who received FUS BBB opening.

	Plasma concentration(%ID/cc)													
	^{89}Zr-C4						^{89}Zr-C4-$^{Fc\text{-}MUT}$							
**Mouse Index**	62	63	66	69	612	67	42	45	44	411	61	64	65	68
**Time(min)**														
0.75	73.88	60.83	78.55	61.21	74.48	65.94	86.56	64.85	72.74	86.69	77.84	79.54	74.90	85.77
1.25	74.38	74.36	76.37	51.20	60.22	51.15	85.93	73.54	77.45	54.58	70.28	69.12	68.57	94.40
2	72.40	51.74	73.57	49.48	74.75	67.90	74.89	57.57	66.74	70.36	77.43	66.59	65.42	70.98
3	0.06	54.52	66.43	60.11	78.29	66.62	67.10	50.33	62.07	56.74	67.06	63.04	62.90	66.72
4	82.23	54.55	73.04	65.93	64.41	53.92	78.91	56.33	63.24	55.83	74.59	71.04	75.45	71.51
5	70.24	52.76	72.56	57.87	76.21	63.29	73.35	55.31	60.45	70.36	75.21	65.98	64.39	71.23
6	72.64	56.42	74.00	54.60	70.99	64.79	63.36	58.16	64.33	64.06	68.38	61.81	69.96	69.80
7.5	71.05	65.78	63.47	53.43	72.49	58.61	67.99	49.57	60.15	59.74	73.58	67.69	68.05	71.01
9.5	69.71	56.26	64.39	60.41	65.46	61.19	69.60	44.89	55.05	61.82	68.28	61.64	67.16	60.92
11.5	66.37	66.63	59.77	55.86	72.82	63.30	68.98	45.75	58.71	59.23	67.96	67.19	64.04	65.47
13.5	66.11	54.21	62.17	52.06	68.89	63.10	66.36	42.46	50.02	57.47	67.67	65.32	63.45	59.93
15.5	67.95	59.70	65.41	51.29	64.39	63.98	63.85	47.19	55.91	53.01	65.09	65.07	57.70	57.96
18	55.21	57.09	58.36	48.10	66.66	61.92	70.47	41.05	49.55	52.30	61.60	61.32	66.66	63.47
21	61.71	57.88	52.29	51.07	63.42	58.05	62.17	40.02	47.72	58.26	58.81	56.15	55.98	66.02
24	60.13	58.12	57.76	55.33	60.24	62.57	63.93	39.40	42.48	54.12	56.86	61.61	60.45	64.30
27.5	60.95	64.38	51.61	50.59	59.96	63.97	61.62	38.81	46.45	47.58	60.17	57.15	55.12	58.85
31.5	62.01	61.22	60.98	51.92	64.17	56.75	60.26	40.09	48.52	51.71	60.97	61.75	53.95	57.90
35.5	59.29	58.46	58.45	50.33	59.18	61.76	63.06	38.46	47.40	43.46	58.38	52.97	52.23	61.08
40	53.49	60.08	58.20	48.86	60.90	59.91	58.75	39.75	40.87	46.00	60.73	56.11	54.56	59.65
45	55.71	56.32	53.75	46.48	64.41	54.52	57.29	35.60	49.61	44.22	58.81	59.98	50.61	59.01
50	56.24	64.56	56.82	48.14	57.10	60.85	58.79	38.94	39.33	47.81	59.52	53.11	46.95	58.42
55	56.25	55.37	63.79	48.24	51.32	58.25	55.56	40.07	42.13	48.24	59.65	56.74	58.59	56.42
59	51.19	54.24	52.40	46.77	52.13	55.06	51.78	35.83	41.11	52.41	59.54	56.42	45.73	60.03
301±8	34.79	40.98	41.13	34.86	44.00	38.51	26.27	23.67	24.46	22.22	25.50	25.65	26.25	31.84
1324±9	19.78	18.61	16.61	15.40	16.99	18.92	6.97	6.17	4.43	5.23	6.67	6.58	6.01	4.94
2773±57	14.38	16.12	13.82	12.53	14.42	13.49	3.08	2.21	2.25	1.83	2.69	1.99	2.78	2.26
4199±27	12.63	14.31	11.65			12.80	1.28	1.43	1.60		1.81	1.86	1.34	1.32
9980±30	7.24	10.16	9.49	9.77	7.75	8.95	0.85	0.58	1.11	1.53	0.87	1.09	1.24	1.57

Table S2. Individual concentrations of ^{89}Zr-DFO-C4 and ^{89}Zr-DFO-C4$^{Fc\text{-}MUT}$ in the contralateral hemisphere of mice who received FUS BBB opening.

	Contralateral hemisphere concentration(%ID/cc)													
	^{89}Zr-C4						^{89}Zr-C4-FcMUT							
**Mouse Index**	62	63	66	69	612	67	42	45	44	411	61	64	65	68
**Time(min)**														
0.75	2.49	2.33	2.40	1.93	2.98	2.54	2.64	2.52	2.71	3.29	2.80	2.30	1.98	2.62
1.25	2.12	2.22	2.13	1.86	2.99	2.49	2.65	2.82	2.86	3.21	2.79	2.39	2.11	2.35
2	2.65	2.48	2.21	1.75	3.10	2.75	2.66	2.79	2.60	3.26	2.85	2.36	2.40	2.33
3	2.85	2.42	2.55	1.89	3.01	3.30	2.73	2.64	2.53	3.02	3.08	2.40	2.43	2.57
4	2.88	3.12	2.71	1.95	3.21	3.06	2.89	3.06	2.81	3.43	3.23	2.72	2.61	3.00
5	3.18	3.29	2.89	2.06	3.29	3.54	3.04	2.66	2.81	3.11	3.36	2.64	2.68	3.08
6	3.29	3.28	2.87	1.99	3.25	3.66	3.22	3.10	3.08	3.52	3.55	2.76	2.90	3.49
7.5	3.31	3.15	3.08	1.95	3.44	3.85	3.20	2.91	2.88	3.34	3.55	2.84	2.92	3.45
9.5	3.24	3.28	3.08	1.97	3.46	3.80	3.36	2.90	3.04	3.40	3.66	2.86	3.43	3.63
11.5	3.46	3.60	3.28	1.98	3.69	4.03	3.33	2.97	3.02	3.52	3.76	2.89	4.14	3.63
13.5	3.36	3.83	3.18	2.00	3.60	4.15	3.47	3.09	3.04	3.45	3.78	2.99	4.01	3.85
15.5	3.45	3.84	3.22	1.96	3.69	4.09	3.32	3.04	3.09	3.46	3.93	2.99	3.80	3.70
18	3.53	3.80	3.35	2.01	3.74	4.35	3.31	3.15	2.99	3.48	3.96	2.99	3.88	3.86
21	3.52	4.01	3.35	2.03	3.78	4.39	3.29	3.22	2.94	3.53	3.82	2.87	3.87	3.99
24	3.57	4.10	3.43	1.99	3.78	4.28	3.54	3.23	2.93	3.74	3.99	2.88	3.84	3.91
27.5	3.66	4.27	3.54	2.03	3.82	4.64	3.39	3.17	2.97	3.51	4.13	2.97	3.90	4.11
31.5	3.71	4.35	3.47	2.12	3.86	4.57	3.28	3.23	2.92	3.56	4.07	3.07	3.75	4.25
35.5	3.75	4.36	3.62	1.99	3.83	4.79	3.27	3.17	2.91	3.51	4.20	3.04	3.80	4.29
40	3.71	4.44	3.74	2.04	3.90	4.83	3.30	3.36	2.98	3.64	4.10	3.15	3.67	4.26
45	3.61	4.40	3.78	2.08	3.84	4.87	3.20	3.28	3.00	3.57	4.14	3.02	3.68	4.34
50	3.81	4.62	3.78	2.12	3.91	5.06	3.12	3.21	2.92	3.47	4.24	3.02	3.61	4.43
55	3.81	4.54	3.87	2.07	3.89	5.03	3.17	3.28	2.91	3.60	4.27	3.03	3.73	4.40
59	3.69	4.67	3.95	1.99	3.84	5.10	2.99	3.31	2.73	3.42	4.17	3.08	3.62	4.43
301±8	3.94	4.28	5.01	2.14	4.41	4.54	3.98	2.93	2.87	3.08	4.05	2.54	2.75	3.74
1324±9	2.19	2.71	3.41	1.23	0.64	2.38	1.58	1.47	1.51	1.67	2.13	1.26	1.30	2.04
2773±57	1.40	1.90	2.54	1.03	0.59	1.70	1.14	0.97	1.10	1.25	1.41	0.91	0.93	1.38
4199±27	1.27	1.67	2.01			1.49	0.91	0.86	0.87		1.18	0.86	0.80	1.28
9980±30	1.48	1.45	1.83	1.31	1.53	1.33	1.07	0.75	0.86	1.21	1.05	0.79	0.80	1.20

Table S3. Individual concentrations of ^{89}Zr-DFO-C4 and ^{89}Zr-DFO-C4$^{Fc\text{-}MUT}$ in the tumor enhanced on post-contrast T1-weighted MRI of mice who received FUS BBB opening.

	Tumor T1w MRI contrast enhanced concentration (%ID/cc)													
	^{89}Zr-C4						^{89}Zr-C4-FcMUT							
**Mouse Index**	62	63	66	69	612	67	42	45	44	411	61	64	65	68
**Time(min)**														
0.75	1.74	1.36	1.02	2.01	2.28	1.27	3.50	1.51	1.42	1.94	1.98	1.94	2.51	1.66
1.25	2.12	1.99	3.93	3.32	1.59	1.69	2.44	1.54	1.18	2.54	2.08	2.08	2.02	3.04
2	2.10	2.45	2.80	2.56	1.71	2.39	2.55	1.95	1.98	2.69	1.61	2.26	1.94	2.31
3	1.87	2.39	3.19	2.11	2.05	1.81	2.65	1.94	2.03	2.47	2.18	2.48	2.09	1.95
4	2.19	2.37	2.98	3.73	2.27	2.29	2.35	2.19	2.51	2.99	2.64	2.71	2.90	2.41
5	2.39	2.60	3.42	2.56	1.71	2.33	3.31	2.22	2.74	3.30	2.72	2.66	2.43	2.82
6	2.45	2.81	3.80	2.89	2.41	2.04	3.33	2.56	2.82	3.46	3.22	2.63	2.45	2.83
7.5	2.72	2.84	3.48	2.73	2.66	2.66	3.26	2.94	2.85	3.19	3.14	2.80	2.08	2.74
9.5	2.92	2.87	4.48	3.17	2.72	2.22	3.21	3.02	3.00	3.37	2.96	2.97	3.16	3.06
11.5	3.24	3.63	4.10	2.69	2.22	2.15	3.77	3.37	3.52	3.51	3.61	3.04	5.04	2.98
13.5	3.12	3.09	4.05	2.88	2.51	2.31	3.58	3.32	3.06	3.33	3.29	3.41	3.88	3.04
15.5	3.47	2.97	3.68	2.75	2.52	2.36	3.78	3.65	3.67	3.33	3.35	3.37	3.71	2.96
18	3.49	3.48	4.26	3.42	2.30	2.64	3.92	3.51	3.80	3.61	4.41	3.46	3.67	2.99
21	3.67	3.68	4.10	4.02	2.41	2.38	4.80	3.70	3.58	3.82	3.92	3.43	3.46	3.58
24	3.74	3.64	4.74	3.56	2.46	2.87	4.81	4.07	3.74	4.15	3.88	3.52	3.04	3.19
27.5	3.51	3.71	5.19	2.88	2.69	2.50	4.42	4.45	3.79	4.07	4.40	3.65	3.09	3.68
31.5	3.71	3.83	5.23	3.44	2.69	2.70	5.11	4.22	4.11	4.35	4.19	3.64	3.12	3.64
35.5	3.90	3.88	5.25	3.99	2.57	3.08	5.05	4.57	3.66	4.49	4.06	3.57	2.95	3.54
40	4.26	4.11	5.70	3.25	2.80	2.77	5.08	4.51	3.72	4.51	4.94	4.07	2.94	3.16
45	4.47	4.24	4.91	3.47	3.08	3.12	5.55	4.23	4.25	4.67	4.73	3.68	2.84	3.65
50	4.50	4.13	5.88	3.67	3.05	3.02	4.97	4.27	4.32	4.65	4.79	4.08	2.87	3.75
55	4.29	4.28	5.41	4.00	2.73	2.86	5.94	4.56	4.02	4.46	5.31	4.02	2.54	3.73
59	4.44	3.97	6.10	4.35	2.57	3.30	5.86	4.76	4.26	4.58	4.69	4.06	3.10	3.40
301±8	5.36	4.71	5.92	4.27	3.03	3.36	7.01	4.63	4.54	6.67	6.59	4.16	2.99	4.15
1324±9	5.51	3.00	3.89	4.62	0.52	2.80	4.86	2.61	2.33	5.18	4.48	3.09	2.13	4.02
2773±57	6.39	2.26	3.31	5.86	0.32	2.34	3.53	1.81	1.75	4.35	3.52	2.46	1.54	3.20
4199±27	7.14	2.14	2.94			2.57	2.88	1.65	1.59		2.97	2.31	1.32	2.76
9980±30	8.53	2.61	7.05	12.31	4.74	2.37	1.32	0.76	0.84	1.84	1.83	1.44	0.98	2.19

Table S4. Individual concentrations of ^{89}Zr-DFO-C4 and ^{89}Zr-DFO-C4$^{Fc\text{-}MUT}$ in the tumor enhanced on PET images of mice who received FUS BBB opening.

	**Tumor PET contrast enhanced (without T1w MRI CE VOI) concentration(%ID/cc)**													
	**^{89}Zr-C4**						**^{89}Zr-C4-FcMUT**							
**Mouse Index**	62	63	66	69	612	67	42	45	44	411	61	64	65	68
**Time(min)**														
0.75	1.75	1.82	1.61	2.49	2.09	2.11	3.09	2.05	2.05	2.16	2.21	2.04	2.75	2.19
1.25	2.11	1.90	3.10	3.21	2.26	2.34	2.86	2.36	2.09	2.12	2.16	2.30	2.79	1.98
2	2.23	2.20	2.42	2.63	1.81	2.79	2.60	2.16	2.41	2.68	2.10	2.14	2.68	2.60
3	2.19	2.20	2.44	2.32	1.99	2.73	2.80	2.66	2.57	2.67	2.16	2.50	3.01	2.74
4	2.44	2.21	2.64	2.70	2.21	3.23	3.06	2.72	2.90	2.67	2.79	3.05	3.47	3.27
5	2.65	2.31	2.90	2.92	2.22	3.22	3.21	2.65	3.05	3.10	2.88	2.83	3.39	3.10
6	2.63	2.49	3.38	2.85	2.53	3.01	3.35	2.80	3.14	3.74	2.93	2.84	3.19	3.04
7.5	2.82	2.84	3.44	2.98	2.54	3.37	3.33	3.21	3.43	3.12	3.21	2.92	3.45	3.70
9.5	2.85	2.77	3.58	2.86	2.65	3.56	3.21	3.15	3.43	3.44	3.24	3.29	4.29	3.69
11.5	2.91	3.47	3.68	2.95	2.23	3.96	3.48	3.35	3.51	3.48	3.30	3.31	5.73	3.90
13.5	3.14	2.87	4.08	2.96	2.60	4.18	3.60	3.26	3.74	3.69	3.62	3.76	5.27	3.79
15.5	3.00	3.10	3.68	2.86	2.59	3.98	3.72	3.53	3.93	4.32	3.47	3.27	5.19	3.99
18	3.15	3.33	4.18	3.11	2.42	4.50	3.76	3.61	3.90	3.99	3.81	3.22	5.30	4.30
21	3.47	3.46	4.34	3.29	2.55	4.69	3.98	3.53	3.91	4.28	3.95	3.63	4.96	4.09
24	3.39	3.46	4.31	3.50	2.66	4.39	4.02	3.68	3.90	3.94	4.17	4.00	4.93	4.21
27.5	3.49	3.69	4.64	3.06	2.67	4.90	4.06	3.77	4.02	4.33	4.21	3.74	4.70	4.55
31.5	3.70	3.71	4.95	3.27	2.67	4.98	4.20	3.84	4.18	4.57	4.23	3.55	4.63	4.61
35.5	3.67	3.69	5.18	3.45	2.64	4.95	4.31	4.06	4.08	4.59	4.27	3.86	4.65	4.44
40	3.77	4.07	5.29	3.23	2.83	5.13	4.45	4.14	4.22	4.86	4.59	4.02	4.60	4.66
45	3.90	3.90	4.95	3.29	2.88	5.40	4.77	4.00	4.33	5.00	4.81	3.97	4.42	4.84
50	3.67	3.92	5.40	3.76	2.95	5.37	4.63	3.93	4.21	4.91	4.83	4.14	4.41	5.02
55	3.89	4.13	5.52	3.63	3.01	5.46	4.68	4.07	4.28	5.03	4.92	4.00	4.37	5.26
59	3.74	3.78	5.19	3.70	2.87	5.47	4.90	4.08	4.21	4.88	4.45	4.16	3.87	5.01
301±8	4.23	4.73	6.67	3.66	2.93	5.48	5.30	4.50	4.28	5.92	5.70	4.48	3.44	5.20
1324±9	3.79	2.67	4.17	3.17	0.82	2.93	3.66	2.50	2.34	4.42	3.97	3.25	1.46	3.28
2773±57	4.27	2.07	3.37	3.90	0.50	2.33	2.50	1.60	1.60	3.22	3.03	2.37	0.95	2.43
4199±27	4.80	1.89	3.06			2.35	2.15	1.31	1.29		2.55	2.36	0.88	2.05
9980±30	7.85	2.36	6.41	9.07	4.19	3.96	1.54	0.88	0.98	1.65	1.63	1.19	0.77	1.50
